# Microbial ecosystem assessment and hydrogen oxidation potential of newly discovered vent systems from the Central and South-East Indian Ridge

**DOI:** 10.3389/fmicb.2023.1173613

**Published:** 2023-10-11

**Authors:** Nicole Adam-Beyer, Katja Laufer-Meiser, Sebastian Fuchs, Axel Schippers, Daniela Indenbirken, Dieter Garbe-Schönberg, Sven Petersen, Mirjam Perner

**Affiliations:** ^1^Marine Geosystems, GEOMAR Helmholtz Centre for Ocean Research Kiel, Kiel, Germany; ^2^Federal Institute for Geosciences and Natural Resources (BGR), Hannover, Germany; ^3^Leibniz Institute of Virology, Hamburg, Germany; ^4^Institute of Geosciences, Christian-Albrechts-Universität zu Kiel, Kiel, Germany; ^5^GEOMAR Helmholtz Centre for Ocean Research Kiel, Kiel, Germany

**Keywords:** 16S rRNA, geochemistry, hydrothermal vents, sediments, Indian Ridge, enrichment, hydrogen oxidation

## Abstract

In order to expand the knowledge of microbial ecosystems from deep-sea hydrothermal vent systems located on the Central and South-East Indian Ridge, we sampled hydrothermal fluids, massive sulfides, ambient water and sediments of six distinct vent fields. Most of these vent sites were only recently discovered in the course of the German exploration program for massive sulfide deposits and no previous studies of the respective microbial communities exist. Apart from typically vent-associated chemosynthetic members of the orders *Campylobacterales*, *Mariprofundales*, and *Thiomicrospirales*, high numbers of uncultured and unspecified Bacteria were identified via 16S rRNA gene analyses in hydrothermal fluid and massive sulfide samples. The sampled sediments however, were characterized by an overall lack of chemosynthetic Bacteria and the presence of high proportions of low abundant bacterial groups. The archaeal communities were generally less diverse and mostly dominated by members of *Nitrosopumilales* and *Woesearchaeales*, partly exhibiting high proportions of unassigned Archaea. Correlations with environmental parameters were primarily observed for sediment communities and for microbial species (associated with the nitrogen cycle) in samples from a recently identified vent field, which was geochemically distinct from all other sampled sites. Enrichment cultures of diffuse fluids demonstrated a great potential for hydrogen oxidation coupled to the reduction of various electron-acceptors with high abundances of *Hydrogenovibrio* and *Sulfurimonas* species. Overall, given the large number of currently uncultured and unspecified microorganisms identified in the vent communities, their respective metabolic traits, ecosystem functions and mediated biogeochemical processes have still to be resolved for estimating consequences of potential environmental disturbances by future mining activities.

## Introduction

Mid-Ocean Ridges, which are formed at spreading centers of tectonic plate boundaries, are the most common setting for the presence and formation of deep-sea hydrothermal vent systems. In these systems, geothermally heated and reduced fluids are emitted through fissures of the ocean floor. By mixing with the cold ambient seawater, minerals precipitate from the hot fluids (reaching temperatures of 400°C or even more) forming the typically observed chimney structures, enriched in various metals (Kelley et al., 2002; Haase et al., 2007). With the aim to achieve a carbon-neutral sustainable economy in the next decades, several metals have become important for implementing the energy and digital transformation. Therefore, deep-sea mining activities have moved into focus (Petersen et al., 2016). Actively venting hydrothermal systems provide a multitude of inorganic electron donors and acceptors (the latter mostly originating from the mixing with ambient seawater), that enable primary biomass production of microorganisms fueling species-rich ecosystems in the deep-sea (Hügler and Sievert, 2011; Dick et al., 2013; Levin et al., 2016). Depending on the geological setting and the host-rocks that shape the chemical fluid compositions, the most abundant electron-donors are hydrogen (H_2_) and/or hydrogen sulfide (H_2_S) (e.g., Wetzel and Shock, 2000; Perner et al., 2007; Adam and Perner, 2018). The availability of the reduced compounds strongly depends on the degree of mixing processes, which result in steep thermal and chemical gradients, providing a dynamic habitat for the prevailing microorganisms (Adam and Perner, 2018 and reference therein).

Microbial communities and their potential roles in biogeochemical cycles and ecosystem functions have been studied at the majority of the known Mid-Ocean Ridges. However, compared to the Mid-Atlantic Ridge (MAR) and East-Pacific Rise (EPR), the Indian Ridge (IR) still remains underrepresented. Microbiological studies at the Indian Ridge have so far included the Kairei, Edmond, and the newly discovered Onnuri vent fields at the slow-spreading Central Indian Ridge (CIR) (Nakamura and Takai, 2015; Han et al., 2018; Adam et al., 2019; Namirimu et al., 2022), the Longqi, Tianzuo and recently identified Old City hydrothermal fields at the ultraslow-spreading South-West Indian Ridge (SWIR) (Li et al., 2016; Ding et al., 2017; Yang et al., 2019; Lecoeuvre et al., 2021) as well as the recently discovered Pelagia vent site along the intermediate-spreading South-East Indian Ridge (SEIR) (Han et al., 2018). Predominantly, these studies have focused on the compositions of the microbial communities based on 16S rRNA gene tags and the putative metabolic functions and environmental implications of the identified taxa.

Starting in 2015, the German Federal Institute for Geosciences and Natural Resources (BGR) launched a program (INDEX) for the exploration of massive sulfide occurrences (SMS) and environmental base line studies in a license area on the Indian Ridge, awarded to Germany by the International Seabed authority. Compared to the MAR, a knowledge deficiency is postulated for IR vent environments regarding the baseline categories required for the determination of mining impacts. This gap includes both the geological background and the microbial and macrofaunal ecosystem functions (Amon et al., 2022). A recent review summarized and analyzed the insights (with respect to putative mining impacts) on IR ecosystems gained so far, but focused almost exclusively on the studies and descriptions of macrofaunal communities, which are known to comprise several endemic species contributing to the need of protection of these ecosystems (van der Most et al., 2023). However, in active vent environments, microbes also fulfill essential ecosystem functions and services like primary production and detoxification of harmful compounds, but also represent a valuable genetic resource for biotechnological or even medical applications (Orcutt et al., 2020 and references therein). For various reasons (including technical and ecological issues) the mining of active hydrothermal vent systems has become unlikely, moving inactive vent sites deposits in the focus of putative mining activities (Koschinsky et al., 2018; Van Dover et al., 2018; Amon et al., 2022). While the role of microbial communities and the possible impacts of their removal from active vent environments are known, the impact and ecosystem services of microbes in inactive vent sites – including element cycling and primary production - are only poorly understood (Orcutt et al., 2020; Van Dover et al., 2020). Since macrofaunal populations are far less abundant in inactive compared to active vents, the understanding of all microbial ecosystem functions in inactive vent environments is an essential prerequisite for the assessment of possible mining effects of SMS deposits.

In the course of the INDEX program, we already performed microbiological studies with sample material from the Kairei and Pelagia vent fields, collected within the framework of the 2016 sampling campaign (Han et al., 2018; Adam et al., 2019, 2021). These studies have addressed the enrichment of autotrophic, hydrogen-oxidizing microorganisms, comparisons of microbial community structures of active and inactive chimneys and the characterization of sediment-inhabiting microbial communities along a hydrothermal gradient. Several distinct phylogenetic groups (e.g., members of *Campylobacterales*, *Nautiliales,* and *Aquificales*) were exclusively found in active chimney habitats, while others (e.g., *Desulfobulbales*, *Thiotrichales,* and *Oceanospirillales*) appeared to belong to the inactive chimney exclusive community (Han et al., 2018). The sediment communities in the Kairei region correlated with sediment depth and showed varying compositions along the hydrothermal transition zone. However, near the active vent, no typical vent-associated chemosynthetic microorganisms were observed in the hydrothermally influenced sediments (Adam et al., 2019).

The aim of the present study is to provide an assessment of the microbial communities and their putative metabolisms and functions in the ecosystems of distinct vent fields of the IR, thereby expanding the baseline knowledge of microbial ecosystems in putative mining areas. Here we report on microbial communities from sediments, massive sulfides, silica mounds and fluids of six different vent sites along the CIR and SEIR, as well as ambient water samples collected in the course of the INDEX2019 sampling campaign. For most of the sampled vent systems, no microbiological studies have been carried out before. Our analyses combine the assessment of the microbial community compositions based on 16S rRNA gene sequencing and enrichments of hydrogen-oxidizing Bacteria with correlation analyses related to geochemical parameters.

## Materials and methods

### Sampling sites and sampling collection

**Figure 1:**
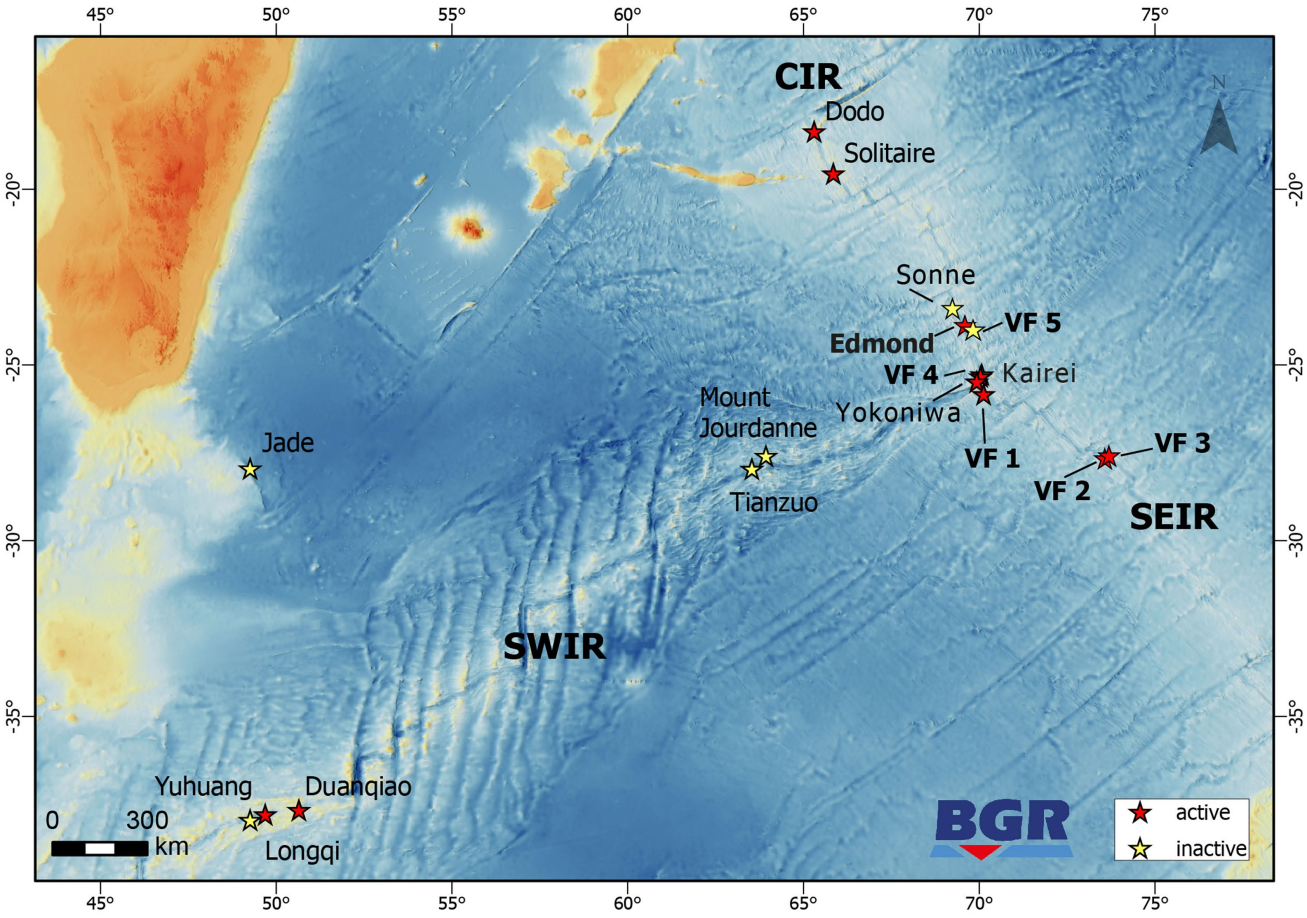
Map showing the locations of inactive and active hydrothermal vent sites along the CIR, SWIR, and SEIR. Locations marked in bold denote sites where microbiology studies have been performed in the framework of the INDEX2019 sampling campaign.

Hydrothermally influenced and reference samples were taken with the help of the remotely operated vehicle (ROV) ROPOS (for technical description refer to https://www.ropos.com) during the INDEX2019 cruise based on the RV Sonne. The sampling sites span six different hydrothermal vent systems along the Central Indian Ridge (CIR) and South-East Indian Ridge (SEIR, see Figure 1 and Supplementary Figure S1). Fluid samples were retrieved by means of the BGR-owned Kiel Pumping System (KIPS, Enwave GmbH), attached to the ROV as previously described (Garbe-Schönberg et al., 2006). Geological sulfide and rock samples were directly collected from the seafloor or snapped from chimney structures. Bacterial mat material was sampled with a slurp gun. Reference water samples were taken in Niskin bottles attached to the ROV or with a conductivity, temperature and depth (CTD) sensor package equipped with a water sampling rosette. Sediment cores were taken with push core liners, operated by the ROV. Two sediment cores, each for microbiological and sediment/porewater analyses, were taken in close proximity to each other. Upon arrival on deck, fluid and water samples were concentrated on polycarbonate membrane filters (0.2 μm pore size; Merck Millipore, Burlington, MA, USA) for microbiological analyses in the home laboratory. Subsamples of fluids were stored at 4°C for later setups of enrichment cultures as described below. For rock and chimney samples, little pieces of rock were broken off using an ethanol-sterilized spatula. This was possible due to the porous and fragile texture of the samples. From the slurp gun sample, flock material was collected by pipetting, using sterile cut-off pipette tips. Using ethanol-sterilized tools, sediment cores were subsampled in 2 cm horizons. All samples for DNA/RNA extractions were frozen at –80°C.

### Porewater sampling and analyses

Porewater was sampled using rhizons (CSS, 5 cm Rhizosphere Research Products B.V., Netherlands) in specially prepared push core liners with a spacing of 2 cm, as described in a previous study (Adam et al., 2019). Minor and trace element concentrations of diluted porewater samples was determined by high resolution ICP-SF-MS (Element XR, Thermo Scientific, Waltham, MA, USA). Details on the sample preparation and measurement procedures can be found in Garbe-Schönberg (1993) and Adam et al. (2019).

### Mineralogy and geochemistry of sulfide, rock, and sediment samples

The suite of collected geological samples comprises massive sulfides, non-sulfidic hydrothermal precipitates and sediments. The samples were documented on board, cleaned and prepared for mineralogical and geochemical analyses. The sediments in the push cores were subsampled at fixed intervals of 2 cm. All whole-rock analyses were conducted at the Activation Laboratories (Actlabs) Ltd. in Ancaster, Canada that provide routine analytical service for the geology and mining sector worldwide. Measurements of the bulk concentrations of major, minor, trace and ultra-trace elements, as well as sulfur were addressed by a combination of analytical packages involving commonly applied techniques, such as ICP-MS, ICP-OES, neutron-activation and infrared spectroscopy. On the basis of geochemical compositions, the normative abundance endmember components were calculated for the sediment samples, reflecting the specific mineral assemblages and their different origins (e.g., pelagic, carbonates component or hydrothermal, sulfidic components). Polished, uncovered, epoxy-embedded thick sections were prepared from representative solid samples (e.g., chimneys) to further study their petrography and mineralogy. The thick sections were investigated using a LEICA DVM6 M digital microscope and a FEI F650 MLA-SEM (scanning-electron microscope equipped with a mineral liberation analyzer), both located at the Federal Institute for Geosciences and Natural Resources (BGR).

### Fluid geochemistry

All hydrothermal fluids were collected using the remotely controlled inert flow-through fluid sampling system BGR-KIPS mounted underneath ROV ROPOS. Immediately after recovery, the fluids were homogenized in the samples flasks, and aliquots taken and filtered for (i) rapid on board analyses, and (ii) subsequent analyses in the home labs on shore. The *ex situ* pH (at 25°C), Eh, conductivity and H_2_S content were measured with suitable electrodes (using a standard approach) in the ship’s labs. The major and minor elements were analyzed with ICP-optical emission spectrometry (Spectro Ciros SOP, Amatek), and trace elements with a ICP-SF-MS (Element XR, Thermo Scientific) at the Institute of Geosciences at the CAU University Kiel. The instruments were calibrated as described in Garbe-Schönberg (1993) and Adam et al. (2019) and data validated using appropriate reference materials.

### Microbial enrichment cultures

Directly after sampling, enrichment cultures were set up with fluid samples in 15 mL Hungate tubes, closed with butyl stoppers and screw-caps. From each fluid sample, 5 mL of fluid were added to 5 mL of organic-free MJ medium as previously described (Hansen and Perner, 2015). The enrichments contained H_2_ as electron donor and NaNO_3_ (5 mM), MnO_2_ (powder), Fe(III) (5 mM, ferrihydrite), SO_4_^2−^ (13.8 mM from MJ medium), or S^0^ (powder) as electron acceptor. To all incubations, except the SO_4_^2−^ reducing ones, 20 mM NaMoO_4_ was added to inhibit sulfate reduction (Oremland and Capone, 1988). The ferrihydrite was synthesized according to Schwertmann and Cornell (2008). The headspace consisted of H_2_/CO_2_ (80/20) for anoxic incubations or H_2_/CO_2_/O_2_ (79/20/1) for microoxic incubations. The incubations were done at room-temperature in the dark. All enrichments contained resazurin as redox indicator. The microoxic incubations were regularly flushed with H_2_/CO_2_/O_2_ once the medium turned clear to re-supply oxygen. All enrichments were transferred several times and activity was confirmed by color change of the resazurin from blue to purple and clear.

To prove the H_2_-consumption activity of the enrichments, we performed experiments where we measured H_2_ in the headspace and respective electron-acceptor consumption or buildup of products from electron acceptor reduction in the medium. To increase the volume of the headspace, these experiments were performed in 100 ml serum vials with 45 ml medium and 5 mL inoculum. The headspace consisted of H_2_/N_2_/CO_2_ (2/78/20) for anoxic enrichments and H_2_/N_2_/CO_2_/O_2_ (2/77/20/1) for microoxic enrichments. Measurements were done directly after inoculating the cultures and after the resazurin showed a color change. Sterile MJ with the respective headspace and electron acceptors added was used as controls. H_2_ concentrations were measured with a Trace GC Ultra gas chromatograph (ThermoFisher Scientific, Waltham, MA, USA), using a ShinCarbon ST 100/120 column (Restek Corporation, Bellefonte, PA, USA) and a Pulsed Discharge Detector (Vici Valco Instruments, Houston, TX, USA) as described before (Hansen and Perner, 2015). Concentrations of NO_3_-were measured according to Schnetger and Lehners (2014) on filtered samples. Sulfide was quantified with the cline assay (Cline, 1969) on samples fixed with 5% zinc acetate. Fe(II) and total Fe concentrations were determined spectrophotometrically with the ferrozine assay (Stookey, 1970) on samples acidified with HCl (1 M final concentration). For total Fe concentrations all Fe(III) was reduced to Fe(II) with hydroxylamine hydrochloride before the assay. Fe(III) was calculated from the difference between Fe(II) and total Fe concentrations. Dissolved Mn(II) was measured with the formaldoxime assay (Brewer and Spencer, 1971). All spectrophotometric assays were performed on 96 well plates with a plate reader (SPECTROstar Nano, BMG Labtech).

### DNA extraction and 16S amplicon sequencing

DNA isolation and 16S amplicon sequencing were performed as previously described (Han et al., 2018; Adam et al., 2019). Briefly, *ca.* 500 mg of rock/chimney/mat material or half of a filter were used for DNA extractions with the Nucleospin DNA Soil Kit (Macherey Nagel, Düren, Germany) according to the manufacturer’s instructions. Bacterial 16S rRNA gene amplicons (V3-V4 region) were generated with the Bact_341F/Bact_805R primer pair, while archaeal amplicons targeting the V4-V5 region were amplified with both the Arch_524F/Arch958R and Arch_519F/Arch_915R primer pairs. All primers contained the Illumina adaptor overhangs and sequencing libraries were prepared using the Nextera Index Kit (Illumina, St. Diego, USA) according to the manufacturer’s recommendations. After a quality and concentration check, a sample pool with equimolar amounts of DNA was sequenced in a 2 × 300 bp paired-end sequencing run on Illumina’s Mi-Seq platform (Adam et al., 2019).

### Sequence data retrieval for comparative analyses

For comparative analyses, sequence raw reads of previous 16S amplicon studies from hydrothermally influenced environments of the Mid-Atlantic Ridge (MAR), CIR and SEIR (both sampled in the course of the INDEX2016 cruise) as well as the South-West Indian Ridge (SWIR) were retrieved from public databases. To ensure the comparability of the datasets, only bacterial sequences of the V3-V4 region were included. Datasets of the following accession numbers and studies were used: SRP503162 (MAR fluids, Gonnella et al., 2016), SRP120106 (INDEX2016 chimney, water and fluid samples, Han et al., 2018), PRJNA474182 (INDEX2016 sediments, Adam et al., 2019) and PRJNA558519 (SWIR sediments, Yang et al., 2019).

### Sequence processing and statistical analyses

Demultiplexed raw sequences from the Illumina MiSeq run as well as the downloaded bacterial raw sequences were quality-checked and processed using the Qiime2 environment (Bolyen et al., 2019). The sequence reads were filtered and merged using the dada2-plugin with default settings, removal of the primer sequences and a trimming of the single raw sequence read length to 260 nucleotides for bacterial sequences prior to merging (Callahan et al., 2016). Taxonomic assignments were performed using a pretrained classifier, based on SILVA database release 138 (Quast et al., 2013), which was trained with the respective primer pairs for Bacteria and Archaea (Pedregosa et al., 2011; Bokulich et al., 2018). The feature-classifier plugin (classify sklearn) was used for taxonomic assignments with default settings and the pre-trained SILVA classifier (Bokulich et al., 2018). After the taxonomic classification, any contaminating sequences were removed from the individual samples: eukaryotic and chloroplast sequences were removed from all datasets, as well as bacterial reads from the archaeal files and vice versa. Sequence alignments and phylogenetic trees were calculated using the “align-to-tree-mafft-fasttree” pipeline (Price et al., 2010). Principle Coordinate Analysis (PCoA) based on weighted and unweighted UniFrac distances and differential abundance analysis (RDA) were performed in R: A language and environment for statistical computing. R Foundation for Statistical Computing, Vienna, Austria. URL https://www.R-project.org/using the microeco package (Liu et al., 2021). Rarefication for PCoA analyses was performed with different subsample sizes: (i) with a sample size 14,000 for the 2019 bacterial sequences, (ii) with 200 subsamples for the 2019 archaeal sequences and (iii) with a sample size of 10,000 sequences for the comparison to sequence data of older studies. Clustering of samples was performed using a confidence level of 0.9. Porewater data used for RDA analyses can be found in Supplementary Table S1.

## Results and discussion

### Geological setting of the sampled vent sites

Samples have been retrieved from three vent fields at the CIR [Edmond, and the yet undescribed Vent Fields (VF) 5 and VF 4] and three fields from the SEIR (VF 1, VF 2 and VF 3; all of which have not been described yet) (see Figure 1 and Table 1). The active Edmond and the neighboring inactive VF 5 hydrothermal fields are both located off-axis and associated with prominent west-facing faults on the eastern flank of the CIR. The associated host rocks are basaltic in composition. The Edmond field comprises numerous active and inactive sulfide chimneys covering an area of more than hundred meters in length and in water depths of 3,330 to 3,250 m (Van Dover et al., 2001). The chlorine-rich, high-temperature fluids (Tmax = 382°C; Gallant and Von Damm, 2006) vent from black smoker chimneys ranging from only a few meters in height to about 35 m-tall, multi-spired structures. The presence of numerous old and disintegrated sulfide structures and abundant sulfide talus indicates long-lasting hydrothermal activity at this site (Van Dover et al., 2001). The inactive VF 5 consists of several mounds (each >200 m in diameter) with multiple standing and toppled chimney structures reaching up to 17 m in height (Müller et al., 2018). Mineralization occurs along a steep, west-facing fault, running parallel to the Edmond fault, at water depths between 3,070 and 3,200 m (Schwarz-Schampera, 2019; Fuchs et al., in prep.).

**Table 1:** Overview of analyzed samples and parameters.

ID	Sample	Type	Vent site	Min. distance to vent (m)	Bac/Shannon index	Arch/Shannon index	PW chemistry	Fluid chemistry	Mineralogy
F2	083ROPOS-KIPS-A	Fluid	VF 1		+/6.96	+/4.74	−	+	−
R1	083ROPOS-F	Sulfide block	VF 1	9	+/6.26	+/5.3	−	−	+
R2	083ROPOS-G	Sulfide chimney	VF 1	3	+/8.15	+/1.76	−	−	+
S3	083ROPOS_PC2-1	Sediment horizon	VF 1	80	+/8.00	+/4.29	+ (PC3)	−	−
S4	083ROPOS_PC2-2	Sediment horizon	VF 1	80	−	+/n.a.	+ (PC3)	−	−
S5	083ROPOS_PC2-3	Sediment horizon	VF 1	80	+/8.52	+/4.09	+ (PC3)	−	−
S6	083ROPOS_PC2-4	Sediment horizon	VF 1	80	+/n.a.	+/n.a.	+ (PC3)	−	−
S7	083ROPOS_PC2-5	Sediment horizon	VF 1	80	+/6.7	+/3.37	+ (PC3)	−	−
S8	083ROPOS_PC2-6	Sediment horizon	VF 1	80	−	+/1.11	+ (PC3)	−	−
S9	083ROPOS_PC2-7	Sediment horizon	VF 1	80	−	+/1.59	+ (PC3)	−	−
F3	104ROPOS-KIPS-C/D	Fluid	VF 2		+/7.51	−	−	+	−
R3	104ROPOS-E	Silica	VF 2	36	+/n.a.	+/1.48	−	−	+
F4	106ROPOS-KIPS-B	Fluid	VF 2		+/8.37	+/3.23	−	+	−
R4	127ROPOS-D	Sulfide chimney	VF 3	3	+/6.8	+/5.66	−	−	+
S10	127ROPOS_PC3-6	Sediment horizon	VF 3	160	+/6.77	−	+(PC2)	−	−
M3	127ROPOS_SG1	Microbial mat	VF 3	14	+/8.18	+/4.81	−	−	−
M2	036SG6	Microbial mat	VF 4	52	+/8.19	+/4.12	−	−	−
F1	040ROPOS-KIPS-C	Fluid	VF 4		+/3.14	+/3.09	−	+	−
W2	031ROPOS N1	Ambient water IN-W	VF 5		+/7.06	−	−	−	−
S1	031ROPOS_PC2	Bulk sediment	VF 5	2,118	+/8.69	−	−	−	−
S2	031ROPOS_PC5-1	Sediment horizon	VF 5	1,147	+/9.46	−	+ (PC4)	−	−
W1	027ROPOS DNA_5	Ambient water IN-R	Edmond		+/n.a.	−	−	−	−
M1	027ROPOS DNA 6	Microbial mat	Edmond		+/8.35	−	−	−	−

Bac and Arch refer to the Amplicon sequencing of Bacteria and Archaea: +/− indicates if 16S rRNA gene tags are available, n.a. = Shannon index is not available for the respective sample. The ID denotes the designation of the (sub-) samples used for microbiological analyses. Porewater (PW) chemistry was determined for duplicate push cores, sampled adjacently to the microbiology cores and given in brackets in the respective column of the table.

The large VF 4 vent field is located in a very different geotectonic setting. Here, hydrothermal activity occurs on a tectonic massif on the western flank of the CIR, close to the Rodriguez Triple Junction, and is associated with the exposure of mantle and lower crustal rocks (Schwarz-Schampera, 2019; Fuchs et al., in prep.). In contrast to the other studied vent fields, VF 4 is associated with mafic to ultramafic plutonic high rocks comprised of gabbros, harzburgithes and pyroxenites, which show an overall high degree of alteration/serpentinization. Black smoker venting of high-salinity fluids occurs at a number of active vent sites in water depths ranging from 2,625 to 3,020 m and reaches temperatures up to 328°C (Schwarz-Schampera, 2019; Fuchs et al., in prep.). Active and inactive vent sites are scattered along the sedimented, axis-facing slope of the tectonic massif.

The three hydrothermal vent fields sampled along the SEIR are all associated with mid-ocean ridge basaltic host rocks. VF 1 is the smallest of the fields and is associated with a small bounding fault on the western flank of the spreading center. Sulfide mineralization is documented only from a small area in water depths ranging from 2,910 to 2,940 m. Currently only low-temperature, diffuse (31°C) (Schwarz-Schampera, 2019; Fuchs et al., in prep.) fluid venting and associated faunal communities have been documented although the presence of sulfide talus and chimney debris indicates that higher temperatures have been present in the past.

The VF 2 and VF 3 hydrothermal fields are the largest vent fields sampled for this study. They are both associated with a large volcanic plateau on the eastern flank of the ridge axis that is cut by major ridge-parallel rift valley faults. Both hydrothermal fields stretch along large, axis-facing fault scarps with numerous active and inactive vent sites. At VF 3 sulfide mineralization has been documented along a strike length of 2.7 km. Hydrothermal mounds at VF 2 stretch over a long distance, however, individual mounds tend to be smaller than at VF 5. In contrast to the high-salinity fluids expelled at the two vent sites along the CIR (Kairei and VF 4), black smoker style venting at VF 2 and VF 3 is characterized by low-salinity fluids (Schwarz-Schampera, 2019; Fuchs et al., in prep.).

### Mineralogy of rock samples

**Figure 2:**
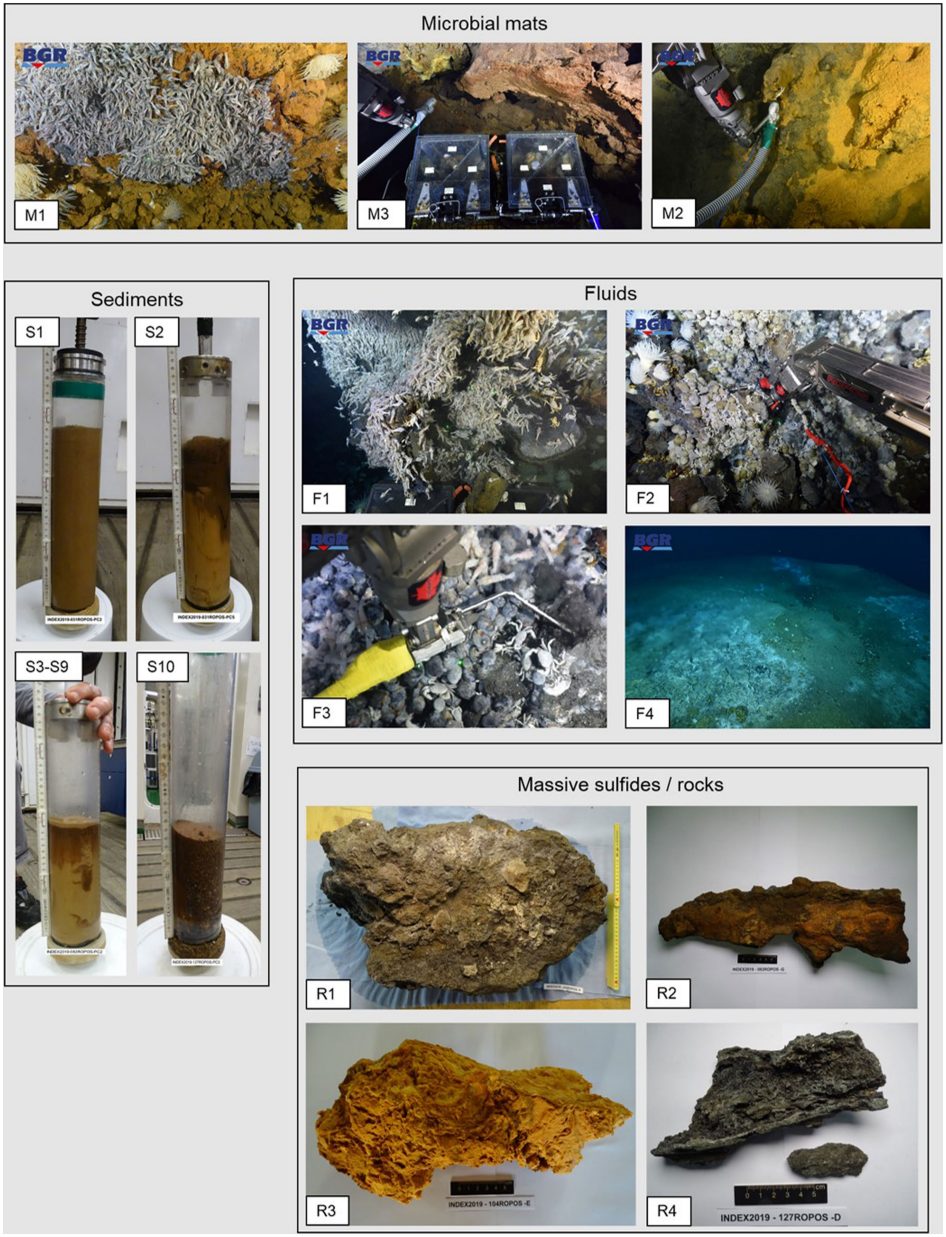
Photos of the sampled microbial mats, fluids, sediments, massive sulfides and rock material. Photos of microbial mats and fluids were taken *in situ* by means of ROPOS’ cameras.

Four hydrothermally-derived sulfidic and non-sulfidic rock samples were collected in the scope of the current study to investigate the relationship between the presence of microbial communities and the rock substrate (Figure 2 and Table 2). The samples 083 ROPOS-F (R1) and 083 ROPOS-G (R2) were collected at the small active VF 1 that vents clear, shimmering fluid. Sample R1 is a large piece of cemented silica, which very likely precipitated in form of sheets from these low-temperature fluids and incorporated abundant talus fragments of pyrite-rich sulfides, pieces of the basaltic host rocks and calcic fossils (e.g., shell fragments of bivalves and gastropods). The other specimen represents the tip of an old, clogged micro-chimney recovered in the vicinity of the active fluid exhaust. The mineralogical study reveals the presence of predominant chalcopyrite and silica, pyrite and small volumes of sphalerite, galena and gold (Table 2). The characteristic concentric zonation of the mineral, with chalcopyrite in the interior, and pyrite, sphalerite and galena to the outside indicates the temperature-dependent sulfide precipitation from previously hot hydrothermal solutions. A white to orange-colored massive piece of silica precipitate (R3) has been recovered from VF 2. It is almost entirely composed of very fine-grained, agglomerated silica particles. More densely-packed silica particles occur in bands that alternate through the sample; minor amounts of very small grains of Fe-oxyhydroxide minerals within the silica are giving the characteristic coloration (Table 2). This specimen originates from Si-rich and metal-poor condensed vapor-phase fluids. A wall fragment of an active black smoker (R4) was collected from VF 3. It consists of numerous micro-sized fluid conduits and is composed of pyrite, marcasite and silica. Minor amounts of chalcopyrite and sphalerite were also observed (Table 2).

**Table 2:** Mineral abundances and characteristics of massive sulfide samples.

ID	Sample	Associated vent field	Sample type	Sample characteristics	Mineral abundances									
					*Cpy*	*Icb*	*Po*	*Py/mc*	*Sl*	*Gn*	*Brn*	*Si*	*Ata*	*FeOOH*
R1	083ROPOS-F	VF 1	Massive sulfide block	Hydrothermal precipitate of pyrite and amorphous silica with enclosed lithoclasts and calcic (seashell) fragments	m	m	+	++	t	t		+++		m
R2	083ROPOS-G	VF 1	Massive sulfide chimney	Tip of a clogged sulfide micro-conduit from an active vent. Distinct temperature-depended mineral concentric zonation from the interior to the outside	++			+	m	t	m	++	t	
R3	104ROPOS-E	VF 2	Silica precipitate	White-to orange-colored solid precipitate of “sugary” silica. The sample contains thin alternating bands of more dense silica with glassy appearance. Minor oxidized Fe-bearing particles give distinct coloration.								+++		m
R4	127ROPOS-D	VF 3	Massive sulfide chimney	Wall-fragment of an active smoking chimney. The sample is characterized by numerous small fluid conduits and abundant pyrite and sphalerite.	m			+++	++			m		

ata, atacamite; cpy, chalcpyrite; brn, bornite; FeOx, Fe-oxyhydroxide-bearing minerals; gn, galena; icb, isocubanite; po, pyrrhotite; py/mc, pyrite/marcasite; si, silica; sl, sphalerite. Symbology: +++, dominant; ++, abundant; +, common; m, minor; t = traces.

### Mineralogy and porewater chemistry of sediments

**Figure 3:**
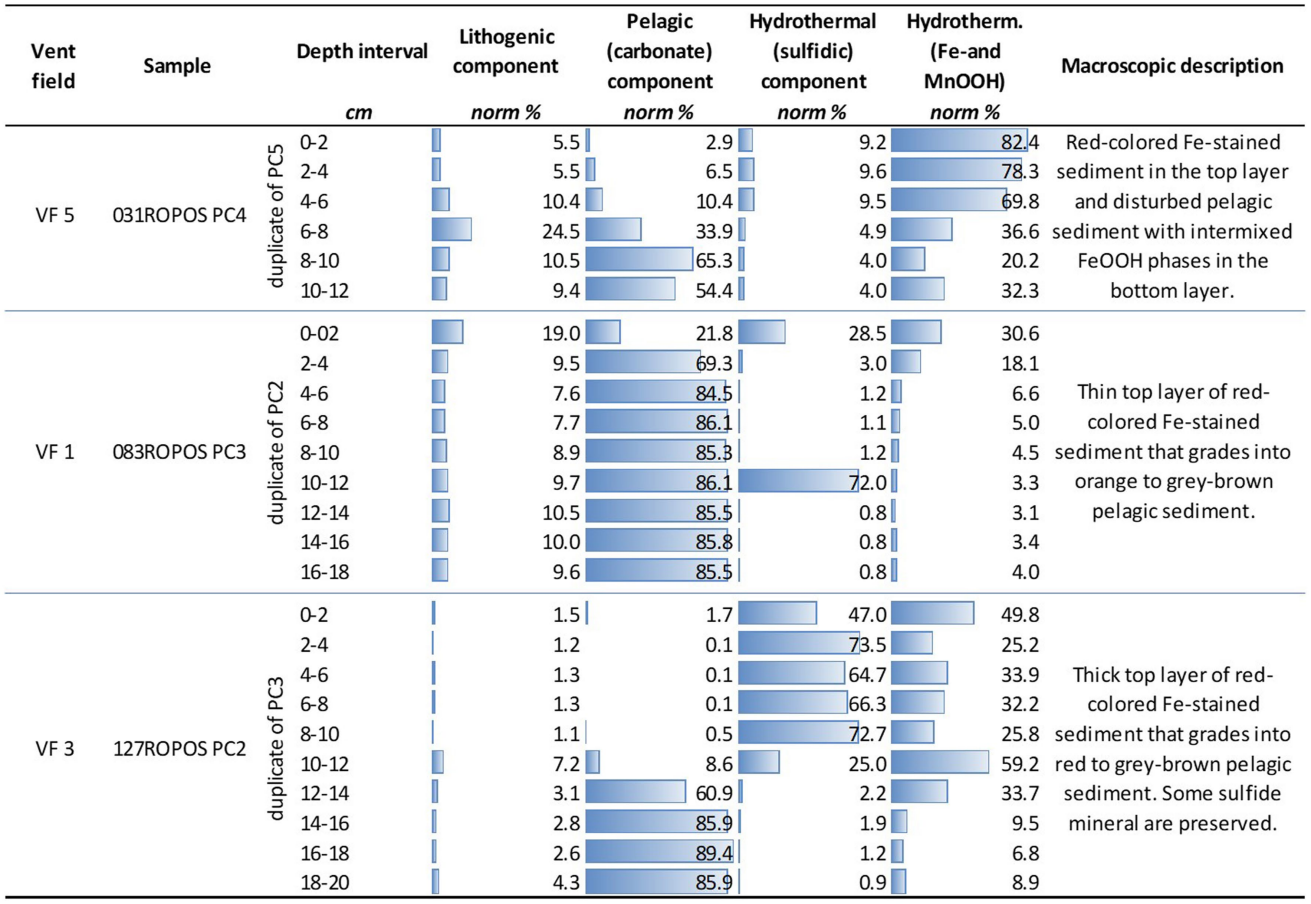
Normative calculation of endmember fractions of sampled push core sediments based on the whole-rock chemistry. The endmembers reflect the abundance of the (i) lithogenic fraction (volcanic and plutonic rock material), (ii) the CaCO_3_-rich pelagic fraction, and (iii) and (iv) the hydrothermal fractions. The latter are subdivided into sulfidic and Fe-/MnOOH mineral-bearing material.

Three push cores were analyzed for their porewater chemistry, and the geochemical and mineralogical composition of the sediments (Figure 3 and Supplementary Table S1). Sample 031ROPOS_PC4 (duplicate of microbiological core 031ROPOS_PC5), collected from VF 5, has a total length of about 15 cm. The uppermost layer is composed of almost 82% of red-colored oxidized and hydrated Fe-minerals of hydrothermal origin and almost 10% sulfides (Figure 3). The high volume of hydrothermal sediment correlated well with increased concentrations of Zn, Pb, Cr, Ni, Mo, and Mn in the porewater (Supplementary Table S1). The hydrothermal components grade into normal carbonate-rich pelagic sediment with increasing depth, that becomes dominant at depths of >8 cm. The push core sample 083ROPOS_PC3 (duplicate of microbiological core 083ROPOS_PC2) from VF1 is characterized by a sulfidic uppermost layer that grades into red-colored Fe-stained sediment, both accumulated during hydrothermal venting. At a depth of about 4 cm and greater, carbonate (background) sediments are predominant (Figure 3). This correlates in turn with a decrease of trace metals (e.g., Pb, Zn, Cu, Cd, Ni, Mn) in the porewater with increasing depth (Supplementary Table S1). The push core 127ROPOS_PC2 (duplicate of microbiological core 127ROPOS_PC-3), collected from the highly active VF 3, contains the highest volumes of sulfides in the top layers, reaching a maximum of 72.7% at a depth of 8 to 10 cm. The hydrothermally-derived Fe-oxyhydroxide and Mn-rich components occur mostly subordinate to the sulfide fraction. There is an abrupt decrease at a depth of 12–14 cm (and greater), where pelagic sediment becomes the predominant fraction (Figure 3).

### Fluid geochemistry

In the scope of this study, we analyzed four low-temperature vent fluid samples from three vent fields. With the exception of 083ROPOS-KIPS-A (F2), the samples are not representing the main (commonly high-temperature) vent fluids (Table 3 and Supplementary Table S2). They are rather collected from slowly venting orifices in vicinity of the main vents, and characterized by low temperatures (max. 14°C) permitting ideal conditions for the settlement of microbial communities. However, fluid chemistry of our diffuse samples can be understood as diluted high-temperature fluid endmembers but all transitional metals needing higher temperatures for transport as dissolved species, e.g., Cu, Co, Ni removed during cooling and mixing with ambient seawater. More details of the corresponding high-temperature vent fluids can be found in Garbe-Schönberg and Schwarz-Schampera (submitted). The chemical analyses of the four hydrothermal samples analyzed in this study showed Mg concentrations, salinities and pH values close to those of normal bottom seawater (Table 3). Regression calculations to zero Mg, standard approach to calculate endmember compositions of the hydrothermal fluids, indeed exhibited a high fraction of seawater in all samples. The hydrothermal fractions in the samples of the slowly venting orifices F1, F3, and F4 contain a hydrothermal fraction of ≤6%; the sample F2 derived from the major vent of VF1 a hydrothermal fraction of 10%.

**Table 3:** Selected physical parameters and chemical compositions of hydrothermal fluid specimen.

ID	Sample	Vent field	Hydrothermal activity	T_max_	pH	EM	Salinity	Eh	H_2_S	Mg	Cl	Si	Fe	Mn	Cu	Zn
				*°C*	at 25°C	[%]	[‰]	mV	μM	mM	mM	mM	μM	μM	μM	μM
F1	040ROPOS-KIPS-C	VF 4	Diffuse shimmering water from beehive	14	7.33	4.96	32.9	–133	*< 10*	51.11	n.a.	0.94	44.70	16.48	*b.d.*	0.06
F2	083ROPOS-KIPS-A	VF 1	Diffuse, clear fluid	31	6.5	10.44	32.3	−251	60	48.16	498.97	1.91	9.44	8.31	*b.d.*	0.02
F3	104ROPOS-KIPS-C/D	VF 2	Diffuse, shimmering water	11,6	7.49	5.49	33.2	−150	23.5	50.83	514.02	0.28	1.29	0.34	0.004	0.01
F4	106ROPOS-KIPS-B	VF 2	Diffuse, shimmering water	< 10	7.27	6.01	33.8	−231	15	50.55	535.02	0.35	n.a.	0.44	*b.d.*	0.03
	Bottom seawater			1.8	7.74	0	31.1	27	0	53.60	532.08	0.01	<1.20	0.61	*<0.002*	0.02

It should be noted that hydrothermal fluids generally contain a significant amount of entrained seawater, as indicated by the hydrothermal endmember proportions (EM). Thus, the presented fluid data reflects compositions of hydrothermal fluid mixed with seawater. b.d.: below the limit of detection, n.a., data not available.

The sample F1 (040ROPOS-KIPS-C) was collected from a beehive structure at the ultramafic rock-hosted VF 4 vent field that vents a diffuse shimmering water of 14°C. This fluid still contained remarkable concentrations of Fe and Mn with 44.7 and 16.48 μM, respectively, in comparison to seawater. The fluid samples F3 and F4, both collected from small fissures exhausting the shimmering fluid, exhibited a similar chemical composition very close to seawater. The fluid F1 (083ROPOS-KIPS-A) was emitted at the vent field VF 1 from a small fissure on top of a basalt talus-covered mound that is characterized by sulfides formed under high-to lower fluid temperatures. The fluid yielded a maximum temperature of only 31°C, indicating the progressive cooling of the hydrothermal system (Schwarz-Schampera, 2019; Fuchs et al., in prep.). In comparison to seawater it contained elevated concentrations of 0.94 mM Si, 9.44 μM Fe and 8.31 μM Mn (Table 3). All of the four investigated fluids still exhibited low Eh values in the range of−133 and-255 mV, and (with the exception of F1) some dissolved H_2_S (up to 60 μM). This provides an indication that reducing condition are maintained and reduced sulfur is still prevalent.

### Taxonomic profiles of microbial communities

The taxonomic profiles of the sampled sediments, fluids, sulfides, rocks and microbial mats broadly resemble community compositions commonly found at deep-sea hydrothermal vent sites (e.g., Flores et al., 2011; Han et al., 2018; Adam et al., 2019). The diffuse fluids show high proportions (7–58%) of *Campylobacterales*, which are typical vent-inhabitants and often dominate microbial vent communities (Adam and Perner, 2018). Generally, the diffuse fluids, as well as rocks and massive sulfides, exhibited smaller proportions of “low abundant” Bacteria (<5% based on order level) than the sediment communities (12–42% compared to 47–72% in sediments). Furthermore, the observed diversities of archaeal communities were significantly lower than those of the bacterial communities, which is also generally reflected by the alpha diversity scores (for details see the section below).

Despite the differing geological settings and partially large distances between the sampled vent systems, no clearly geography-related differences in the taxonomic profiles were observed.

#### Rock communities and microbial mat compositions

Overall, the here analyzed massive sulfide and chimney samples were characterized by large proportions of uncultured and (below the class level) unspecified Bacteria (Table 4), hampering functional and metabolic predictions. Against our expectations, no distinct patterns of similarity between different microbial community compositions were observed with respect to the current hydrothermal activity associated with rock samples, i.e., between active or inactive chimney pieces. The respective bacterial communities of the massive sulfide block R1 (VF 1) and chimney sample R4 (VF 2) showed similar patterns both on the order level and when considering only the 10 most abundant genera with large proportions of Campilobacterota (Supplementary Figure S1A and Table 4), exceeding the relative campylobacterotal abundances previously observed for massive sulfides of the CIR and SEIR (Han et al., 2018). The highest genera-based abundances were observed for the typically vent-associated, chemoautotrophic, sulfur and hydrogen-oxidizing *Sulfurovum* genus (Inagaki et al., 2004; Mino et al., 2014) and the heterotrophic, typically sediment-inhabiting *Carboxylicivirga* genus (e.g., Wang et al., 2016; Table 4). The archaeal communities of R1 and R4 also showed the highest diversities among all analyzed samples with large proportions of *Woesearchaeales* (Supplementary Figure S1B). In the clogged chimney sample R2 we observed large proportions of typically sulfur-oxidizing gammaproteobacterial genera, such as *Thiogranum*, *Thiohalophilus*, and uncultured *Thiotrichaceae* (Sorokin et al., 2007; Chernousova et al., 2009; Mori et al., 2015), adding up to 20% of all observed genera (Table 4 and Supplementary Figure S1). Based on the order level, the bacterial community composition of the clogged chimney piece R2 was similar to that of the VF 1 (surface) sediments (Supplementary Figure S1A), but additionally contained >5% of the deep-sea and hydrothermal vent associated, Fe-oxidizing *Mariprofundales* (Emerson et al., 2007). The archaeal community of R2 was characterized by the highest abundance of *Micrarchaeales* across all samples (Supplementary Figure S1B). Interestingly, no campylobacterotal Bacteria were identified in the silica mound sample from VF 2 (R3), which generally exhibited a quite diverse taxonomic profile. This involved orders frequently found in other sample types like *Planctomycetales*, but also high abundances of orders not found in any of the other samples analyzed in this study (Supplementary Figure S1A). Furthermore, the 10 most abundant genera in this sample were different from those of all other rock samples with large proportions of nitrifying *Nitrosomonas* and *Nitrospina* (e.g., Chain et al., 2003; Lücker et al., 2013), adding up to 13% (Table 4). The high bacterial diversity of the silica sample however, is not reflected in the respective archaeal community, consisting of 100% *Nitrosopumilales*.

**Table 4:** Most abundant bacterial genera of rock and microbial mat communities.

Sample	Sulfide block R1	Sulfide chimney R2	Silica sample R3	Sulfide R4	Microbial mat sample M3	Microbial mat sample M2	Microbial mat sample M1
Site	VF1	VF1	VF2	VF3	VF3	VF4	Edmond
Ten most abundant genera	*Campylobacter* (8%)	*Acidithiobacillaceae* clade 9 M32 (3%)	*Dehalococcoidia* clade SAR202 (8%)	*Campylobacter* (4%)	*Alteromonas* (4%)	*Aliikangiella* (3%)	*Colwellia* (3%)
	*Candidatus* Campbellbacteria (2%)	*Aquibacter* (3%)	NB1-j clade (4%)	*Candidatus* Moranbacteria (4%)	*Jejudonia* (2%)	*Mariprofundus* (20%)	*Mariprofundus* (4%)
	*Carboxylicivirga* (14%)	*Bythopirellula* (4%)	*Nitrosomonas* (5%)	*Carboxylicivirga* (11%)	*Marinobacter* (2%)	*Maritimimonas* (2%)	*Pseudofulvibacter* (3%)
	*Ichthyobacterium* (4%)	*Mariprofundus* (6%)	*Nitrospina* (8%)	*Desulfobulbus* (5%)	*Mariprofundus* (21%)	*Methylomonadaceae* clade pLW-20 (8%)	*Rubritalea* (2%)
	*Nitratifractor* (7%)	*Robiginitomaculum* (3%)	Phycisphaerae clade CCM11a (3%)	*Ichthyobacterium* (6%)	*Mesoflavibacter* (4%)	*Methylomonadaceae* marine methylotrophic group 2 (9%)	*Sulfurimonas* (12%)
	*Oceanithermu*s (3%)	*Thiogranum* (6%)	*Pirellulaceae* Pir4 lineage (5%)	*Nitratifractor* (8%)	*Muricauda* (2%)	*Methyloprofundus* (3%)	*Thiohalomonas* (5%)
	*Sulfurovum* (11%)	*Thiohalophilus* (2%)	*Thermoanaerobaculaceae* Subgroup 10 (3%)	*Oceanithermus* (5%)	*Sulfitobacter* (4%)	*Nitrospina* (2%)	uncultured *Arenicellaceae* (3%)
	uncultured *Ardenticatenales* (2%)	uncultured Gammaproteobacteria (3%)	uncultured *Defluviicoccales* (7%)	*Sulfurovum* (17%)	uncultured *Planctomycetales* (3%)	Phycisphaerae clade CCM11a (2%)	uncultured *Cyclobacteriaceae* (4%)
	uncultured *Saprospiraceae* (2%)	uncultured *Planctomycetales* (3%)	uncultured *Kiloniellaceae* (7%)	uncultured *Saprospiraceae* (5%)	unspecified *Alteromonadaceae* (2%)	*Phycisphaeraceae* clade SM1A02 (2%)	uncultured *Thiotrichaceae* (9%)
	unspecified *Anaerolineaceae* (9%)	uncultured *Thiotrichaceae* (12%)	uncultured *Planctomycetales* (8%)	unspecified Bacteria (4%)	unspecified Gammaproteobacteria (2%)	Planctomycetota clade OM190 (5%)	unspecified *Flavobacteriaceae* (5%)

The ten most abundant genera per sample are displayed with their relative abundance given in brackets.

Although the bacterial (and archaeal) communities of all sampled microbial mats exhibited clear similarities on the order level (Supplementary Figure S1), the 10 most abundant bacterial genera per sample differed almost completely. The only highly abundant genus shared between all microbial mats was the Fe-oxidizing *Mariprofundus*, contributing up to 21% of all genera (Table 4). At the Edmond vent field (M1), mesophilic campylobacterotal and gammaproteobacterial genera potentially involved in sulfur oxidation, i.e., *Sulfurimonas*, *Thiohalomonas*, and uncultured *Thiotrichaceae* (Chernousova et al., 2009; Han and Perner, 2015; Mori et al., 2015), were adding up to 26%. Interestingly, the as obligately psychrophilic described *Colwellia* genus (Techtmann et al., 2016) was also highly abundant in this sample (Table 4). Apart from *Mariprofundus* (20%), methanotrophic Bacteria of two different *Methylomonadaceae* groups and the *Methyloprofundus* genus (Bowman, 2005) constituted a major part of the bacterial community in microbial mat M2 (VF4).

#### Sediment communities

Due to the in most cases limited availability of sediment material and/or sequencing results of the sediments sampled within our study, a comparison of the taxonomic profiles of whole sediment cores of the different vent fields is not possible. However, we were able to identify some patterns in the distribution of certain genera.

The VF 1 sediments (S3–S9), sampled in a distance of *ca.* 80 m to an active vent, were characterized by community shifts with increasing sediment depth, especially regarding the bacterial communities. Apart from the high number of low abundant taxa, *Ectothiorhodospirales*, *Thiotrichales* (both Gammaproteobacteria), and *Pirellulales* (Planctomycetota) dominated the surface layer (0–2 cm) with proportions of more than 10% of Bacteria on the order level (Supplementary Figure S1). The deeper sediment horizons (4–10 cm) were characterized by a core community of *Dehalococcoidia* clade S085 Chloroflexi, Planctomycetota, *Steroidobacterales* and other Gammaproteobacteria in slightly varying percentages. The 6–8 cm horizon additionally contained higher proportions (>5%) of *Dehalococcoidia* clade SAR202 *Chloroflexi*, members of the NBj-1 group as well as members of the *Alphaproteobacteria* (Supplementary Figure S1A). These patterns were largely reflected in the ten most abundant genera (Table 5). However, a high abundance of the sulfate-reducing *Desulfatiglans* (Jochum et al., 2018) in the surface layer as well as increasing abundances of heterotrophic, ubiquitously distributed and sediment-associated *Woeseia* (Hoffmann et al., 2020) (Table 5). The respective archaeal communities showed a much lower diversity and the surface layers (0–4 cm) were characterized by high proportions of unassigned sequences and unspecified Archaea (Supplementary Figure S1B). Such a phenomenon was also observed for sediments of the Kairei vent field (Adam et al., 2019). The predominant archaeal members across all analyzed sediment depths at VF 1 were *Nitrosopumilales* (Nitrososphaera), contributing to up to 100% (in the 6–8 and 10–12 cm horizons, Supplementary Figure S1B). Other *Nitrososphaeria* and *Woesearchaeles* (Nanoarchaeia) contributed to up to 2 and 10%, respectively (Supplementary Figure S1B).

**Table 5:** Most abundant bacterial genera of sediment communities.

Sample	Bulk sediment S1	S2 (0–2 cm)	S3 (0–2 cm)	S5 (4–6 cm)	S6 (6–8 cm)	S7 (8–10 cm)	S10 (10–12 cm)
Site	Score	Score	Surya	Surya	Surya	Surya	Penumbra
Ten most abundant genera	Acidobacteriota subgroup 21 (3%)	*Candidatus* Omnitrophus (5%)	*Desulfatiglans* (5%)	Acidobacteriota Subgroup 22 (3%)	*Blastopirellula* (3%)	*Dehalococcoidia* clade S085 (6%)	*Alphanizomenon* NIES81 (2%)
	*Dehalococcoidia* clade S085 (3%)	*Ignavibacteriales* PHOS-HE36 group (3%)	Gammaproteobacteria clade B2M28 (6%)	*Dehalococcoidia* clade S085 (6%)	*Dehalococcoidia* clade S085 (6%)	NB1-j clade (4%)	*Candidatus* Thiobios (2%)
	*Dehalococcoidia* clade S202 (4%)	Nitrospinota P9X2b3D02 group (3%)	*Pirellulaceae* Pir4 lineage (4%)	*Dehalococcoidia* clade SAR202 (3%)	*Dehalococcoidia* clade SAR202 (5%)	*Phycisphaeraceae* Urania-1B-19 marine sediment group (3%)	*Desulfatiglans* (2%)
	NB1-j clade (4%)	Phycisphaerae clade CCM11a (2%)	*Thiogranum* (17%)	*Methylomirabilaceae* group wb1-A12 (3%)	*Methylomirabilaceae* group wb1-A12 (3%)	Planctomycetota clade OM190 (4%)	*Desulfosarcinaceae* Sva0081 sediment group (5%)
	*Phycisphaeraceae* Urania-1B-19 marine sediment group (3%)	*Pirellulaceae* Pir4 lineage (6%)	uncultured *Anaerolineaceae* (2%)	NB1-j clade (5%)	NB1-j clade (8%)	*Thiogranum* (3%)	*Gammaproteobacteria* clade B2M28 (10%)
	Planctomycetota clade OM190 (3%)	*Schekmanbacteria* (3%)	uncultured *Arenicellaceae* (2%)	*Nitrospira* (3%)	*Phycisphaeraceae* Urania-1B-19 marine sediment group (5%)	uncultured *Defluviicoccales* (4%)	*Thiogranum* (19%)
	uncultured *Arenicellaceae* (3%)	Sva0485 group (3%)	uncultured Gammaproteobacteria (2%)	uncultured *Defluviicoccales* (3%)	uncultured *Defluviicoccales* (6%)	uncultured *Gemmatimonadaceae* (3%)	uncultured *Arenicellaceae* (2%)
	uncultured *Defluviicoccales* (3%)	uncultured *Actinomarinales* (3%)	uncultured *Pirellulaceae* (3%)	uncultured *Kiloniellaceae* (3%)	uncultured *Magnetospiraceae* (5%)	uncultured *Magnetospiraceae* (3%)	uncultured *Chromatiaceae* (2%)
	uncultured *Planctomycetales* (2%)	uncultured *Cyclobacteriaceae* (2%)	uncultured *Thiotrichaceae* (11%)	uncultured *Magnetospiraceae* (4%)	uncultured *Vicinamibacterales* (4%)	uncultured *Planctomycetales* (3%)	uncultured *Pirellulaceae* (2%)
	*Woeseia* (10%)	unspecified Bacteria (17%)	unspecified *Aminicenantales* (3%)	*Woeseia* (7%)	*Woeseia* (6%)	*Woeseia* (10%)	uncultured *Thiotrichaceae* (21%)

The ten most abundant genera per sample are displayed with their relative abundance given in brackets.

Unfortunately, and despite multiple attempts, only one sediment horizon of the core collected in VF 3 yielded bacterial 16S rRNA gene sequences of sufficient quality. This layer (10–12 cm) was characterized by the lowest proportion of low abundant Bacteria on the order level. The most abundant genera were the gammaproteobacterial, sulfur-oxidizing *Thiogranum* and uncultured *Thiotrichaceae* (19 and 21%, respectively, see Table 5). Furthermore, the highest abundances of sulfate-reducing Desulfobacterota were identified in this sediment horizon (*cf.* Supplementary Figure S1 and Table 5).

The two surface sediment samples at VF5 (S1 and S2) showed the highest proportions of low abundant Bacteria on the order level but otherwise differed completely in their community compositions (Supplementary Figure S1A). Interestingly, the bacterial community of bulk sediment sample S1 partially resembled those of deeper sediment horizons of the VF1 sample, exhibiting the highest proportion of *Woeseia* among all surface sediments (Table 5). Sample S2 showed the greatest differences to other sediment samples, sharing only the high abundance of *Pirellulales* with samples S3 and S10 (Supplementary Figure S1A). Also noticeable is the high percentage of unspecified Bacteria (17% of observed genera, see Table 5), which was not observed for any other sample.

#### Microbial communities of diffuse fluids and water samples

Three of the four sampled diffuse fluids (F2, F3, and F4) exhibited similar bacterial community compositions with a clear dominance of *Campylobacterales* (up to 58%, see Supplementary Figure S1A) and high abundances of the gammaproteobacterial *Thiomicrospirales* and *Thiotrichales,* all of which are involved in hydrogen and/or sulfur oxidation and frequently encountered at hydrothermal vents (e.g., Nakagawa and Takai, 2008; Hansen and Perner, 2016; Adam and Perner, 2018). Among these samples the campylobacterotal *Sulfurovum* displayed the most abundant genus with relative proportions between 29 and 42% (Table 6). The respective archaeal communities of fluids F3 and F4 (both from VF2) showed nearly identical compositions, mainly consisting of *Nitrosopumilales* (88–90%), Marine Benthic Group A (Nitrososphaeria, 1–3%) and unassigned archaeal sequences (7–8%, see Supplementary Figure S1B). The archaeal community of F2 however, was characterized by a higher diversity and included high proportions (5%) of Marine Group II Thermoplasmata, which were not observed in any other analyzed sample and are mainly found in ocean surface waters (Rinke et al., 2019). Interestingly, in the ambient water sample retrieved in the realms of the Edmond vent field (W1), the typically vent-associated *Campylobacterales* also appear to dominate the bacterial community (Supplementary Figure S1A). The other major bacterial groups of this less diverse sample were also frequently found in the diffuse fluid samples (Table 6), indicating that a diffuse fluid flow occurred in the vicinity. In addition to the prevailing autotrophic sulfur oxidizers, a notably high abundance of the heterotrophic sulfur-oxidizing genus *Cocleimonas* (Tanaka et al., 2011) was observed in the W1 community (Table 6).

**Table 6:** Most abundant bacterial genera of fluid communities.

Sample	Diffuse fluid F2	Diffuse fluid F3	Diffuse fluid F4	Diffuse fluid F1	Ambient water W2	Ambient water W1
Site	VF1	VF2	VF2	VF4	VF5	Edmond
Ten most abundant genera	*Alcanivorax* (3%)	*Alcanivorax* (7%)	*Alteromonas* (5%)	*Alcanivorax* (56%)	*Alcanivorax* (3%)	*Alteromonas* (2%)
	*Caminibacter* (3%)	*Candidatus* Moranbacteria (2%)	*Dehalococcoidia* clade SAR202 (1%)	*Cocleimonas* (1%)	*Alteromonas* (5%)	*Cocleimonas* (13%)
	*Nitratifractor* (5%)	*Cocleimonas* (2%)	*Marinimicrobia* SAR406 clade (1%)	*Marinobacter* (1%)	*Dehalococcoidia* clade SAR202 (4%)	*Colwellia* (2%)
	*Sulfurimonas* (2%)	*Halomonas* (4%)	SAR324 clade (Marine group B, 1%)	*Mesoflavibacter* (2%)	*Marinimicrobia* SAR406 clade (3%)	JGI_00000069-P22 group (Gracilibacteria, 2%)
	*Sulfurovum* (42%)	JGI_00000069-P22 group (Gracilibacteria, 2%)	*Sulfurimonas* (14%)	*Pseudoalteromonas* (1%)	*Pseudoalteromonas* (10%)	*Pseudoalteromonas* (3%)
	*Thiomicrospira* (6%)	*Nitratifractor* (3%)	*Sulfurovum* (29%)	*Sulfurovum* (7%)	SAR324 clade (Marine group B, 3%)	*Psychromonas* (2%)
	*Thioreductor* (2%)	*Sulfurovum* (34%)	SUP05 cluster (4%)	*Thiomicrospira* (2%)	*Sulfurimonas* (5%)	*Sulfurimonas* (3%)
	uncultured *Thiotrichaceae* (2%)	uncultured Gammaproteobacteria (2%)	*Thiomicrospira* (8%)	uncultured *Cellvibrionaceae* (12%)	SUP05 cluster (16%)	*Sulfurovum* (50%)
	unspecified *Rhodobacteraceae* (3%)	unspecified *Rhodobacteraceae* (4%)	*Thioreductor* (2%)	uncultured *Micavibrionale*s (3%)	unspecified *Alteromonadaceae* (3%)	SUP05 cluster (3%)
	unspecified *Thiotrichaceae* (3%)	unspecified *Sphingomanadaceae* (2%)	uncultured *Arcobacteraceae* (11%)	unspecified *Rhodobacteraceae* (12%)	unspecified *Rhodobacteraceae* (12%)	uncultured *Arcobacteraceae* (2%)

The ten most abundant genera per sample are displayed with their relative abundance given in brackets.

The diffuse fluid of the VF 4 vent system (F1) showed the lowest abundance of *Campylobacterales* and the least diverse bacterial community pattern among the sampled fluids, clearly dominated by the *Alcanivorax* genus (56%, see Supplementary Figure S1A and Table 6). Interestingly, the respective archaeal community broadly resembled those of the other fluids, but displayed the highest number of unassigned sequences observed in this study (Supplementary Figure S1B).

In both the diffuse fluid F1 (VF4) and the ambient water sample W2 (VF5), exceptionally high proportions of uncultured *Rhodospiraceae* (Alphaproteobacteria, 12% each) were observed (Table 6). On the order level, W2 exhibited the highest abundances of *Thiomicrospirales* (18%) and *Alteromonadales* (22%, both Gammaproteobacteria) in this study, but no “ambient-water exclusive” orders were identified (Supplementary Figure S1). The extraction of the 10 most abundant genera revealed an exceptionally high abundance of the gammaproteobacterial SUP05 cluster (16%), which has frequently been found in hydrothermal plume samples and was shown to be involved in sulfur and iron cycling (Hansen et al., 2022; Zhou et al., 2023). However, the typically vent-associated *Campylobacterales* were missing in this sample (Supplementary Figure S1 and Table 6).

### Diversity of microbial communities

Overall, a much higher alpha diversity was observed in the bacterial communities compared to the archaeal ones (Supplementary Figure S1 and Table 1). The respective Shannon indices ranged from 3.1 to 9.5 for Bacteria, while they were limited to 1.1 to 5.7 for Archaea, reflecting the observations of the taxonomic profiles. Likewise, the highest Shannon diversity was observed for bacterial sediment communities (Table 1). Due to the limited number of successfully sequenced sediment samples, a clear zonation of the microbial diversity along the depth of the sediment core could not be detected. Site-specific differences in the Shannon indices could not be observed.

**Figure 4:**
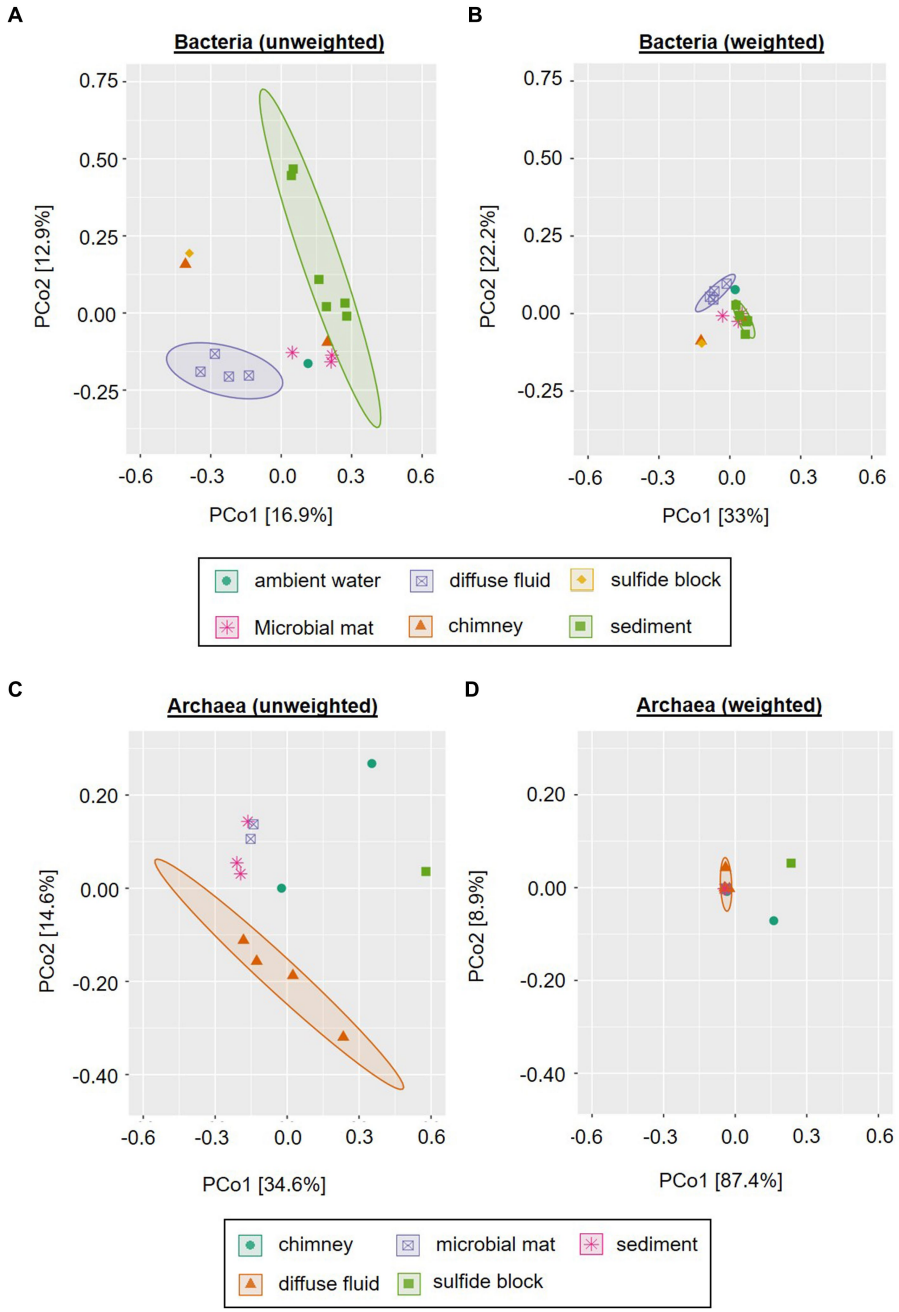
Principle Component Analysis of INDEX2019 samples. Unweighted **(A,C)** and weighted **(B,D)** Unifrac distances for Bacteria **(A,B)** and Archaea **(C,D)** are displayed. Ellipses show clusters based on a confidence level of 0.9 and *n* > 3 per parameter (i.e., sample type). Please note that for reasons of visibility, differing axe sizes were used for Bacteria and Archaea.

In order to assess the beta-diversity of the amplicon-derived microbial communities, we performed principle coordinates analyses (PCoA) with subsets of the bacterial and archaeal 16S gene tags of the 2019 INDEX cruise, using unweighted and weighted Unifrac distances. Due to the cut-off set for these analyses, samples R3, S6 and W1 were excluded from the bacterial plots and samples R3, S4, S6, S8, and S9 from the archaeal plots. The ordination for bacterial communities showed a certain clustering according to the type of sample. This effect was more pronounced in the unweighted compared to the weighted analysis (*cf.* Figures 4A,B). Furthermore, the scattering directions differed slightly and the ordination of the samples along both coordinates occurred with smaller distances between the samples and clusters. This effect again is likely caused by the overall high numbers of low abundant Bacteria in our samples. In both analyses, the chimney sulfide R4 and the sulfide block R1 formed a distinct group, which exhibited a large distance to all other samples (Figures 4A,B). Yet, as these are only two samples they are not marked as a statistically relevant cluster in the plot. The bacterial sediment communities also clustered together quite closely. However, there were two outliers (the VF 1 surface sediment S3 and the 10–12 cm sediment layer S10) in the unweighted analysis, which were separated along coordinate 2. Another cluster was composed of the four diffuse fluids, which was accompanied by the ambient water sample W2 (VF 5) in the weighted analysis (Figure 4 A and B). In the unweighted Unifrac analysis, the latter was positioned among the microbial mats and massive sulfide sample R2, forming a group between the sediment and fluid samples.

Similar to the bacterial communities, the archaeal communities also showed a sample type-specific clustering in the unweighted Unifrac analysis, albeit with differing cluster compositions (*cf.* Figures 4A–D). In contrast to the bacterial, the archaeal sediment sample S3 (surface horizon from VF 1) grouped closely together with microbial mat samples M2 and M3 along coordinate 2. Furthermore, a cluster containing the diffuse fluids extended along coordinate 1. Two samples were separated from the clusters and also showed diverging ordinations among each other: the sulfide block R1 and the sulfide chimney R4 (Figure 4C). In the weighted analyses, the majority of all samples formed a single cluster in the center of the plot (Figure 4D). The three distinctly different samples (diffuse fluid F3, sulfide rock R1 and sulfide chimney R4) however showed diverging ordinations: while F3 was only separated along coordinate 1 and appeared to belong to the cluster according to the applied statistics, R1 and R4 showed distinct ordinations along both axes. Thus, the greatest difference between archaeal communities was observed for the sulfide samples R1 and R4.

**Figure 5:**
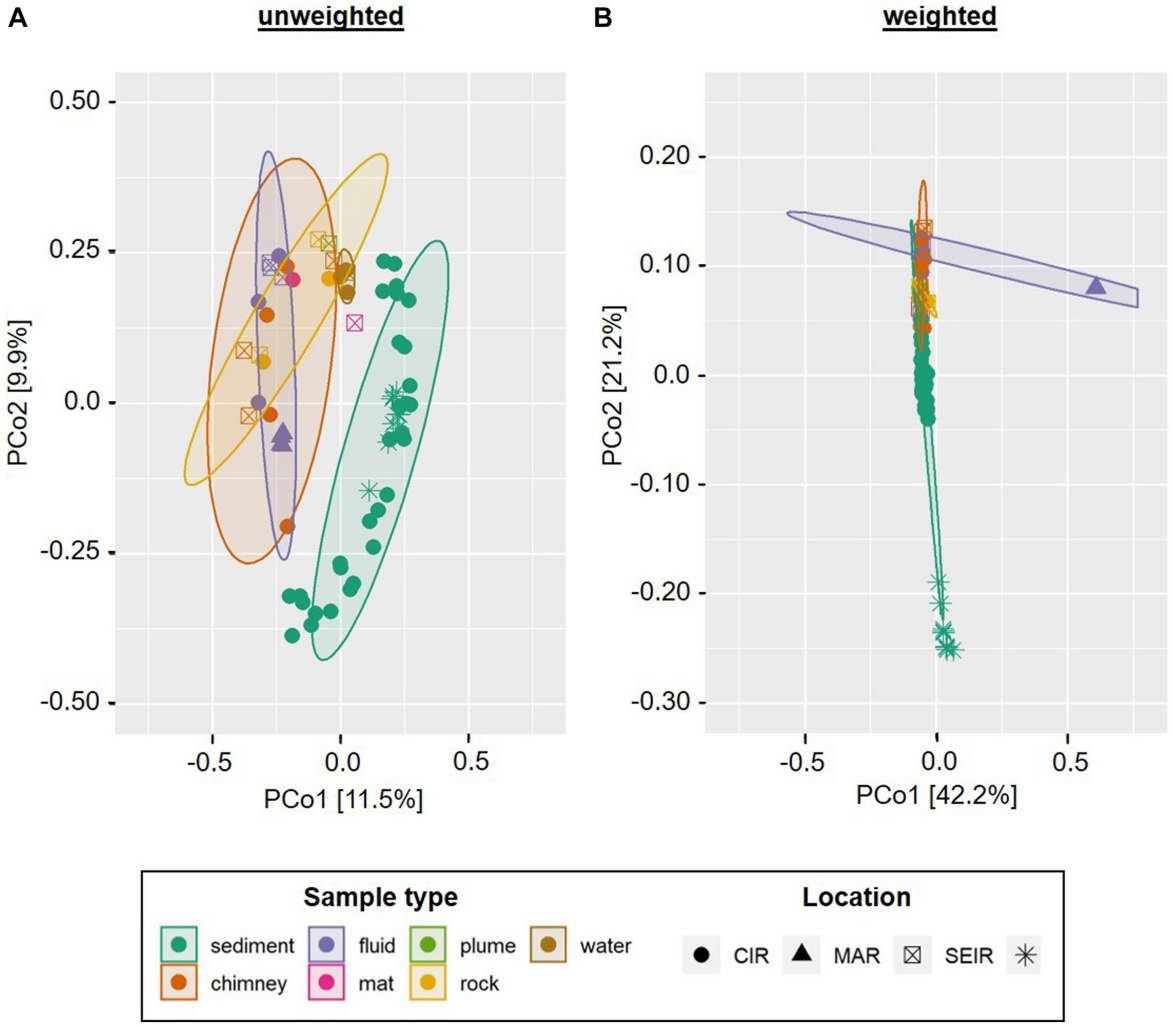
Principle Component Analysis of INDEX2019 samples in comparison with INDEX2016, SWIR sediments and MAR fluids. Unweighted **(A)** and weighted **(B)** Unifrac distances are displayed for bacterial communities only. Ellipses show clusters based on a confidence level of 0.9 and *n* > 3 per parameter (i.e., sample type). Please note that for reasons of visibility, differing axe sizes were used for the weighted and unweighted analyses.

Furthermore, we compared the bacterial communities to those of a former INDEX cruise as well as fluids from the mid-Atlantic Ridge (MAR) and sediments from the South-West Indian Ridge (SWIR). In the unweighted Unifrac analysis the sediment samples of INDEX cruises clustered together with those of the SWIR, clearly separated from the remaining sample types (Figure 5A). The MAR fluids formed a small group within the computed fluid cluster, which still exhibited higher similarities to chimney and rock samples from the CIR and SEIR than to the majority of Indian Ridge (IR) fluids. The latter were closely arranged to ambient water, microbial mat, rock and plume samples (Figure 5A). When the abundance of the taxa is taken into account, the sediment communities of the SWIR formed a distinct cluster separated at a great distance from the other samples along coordinate 2 (Figure 5B). The MAR fluids took another outlier position, while the remaining (IR) samples clustered in the center of the ordination plot with smaller sample type-specific differences. Thus, in the weighted analysis, “Ridge-specific” differences in the beta diversity of bacterial communities dominated (Figure 5B).

### Enrichments of hydrogen-oxidizing microorganisms from diffuse fluids

**Figure 6:**
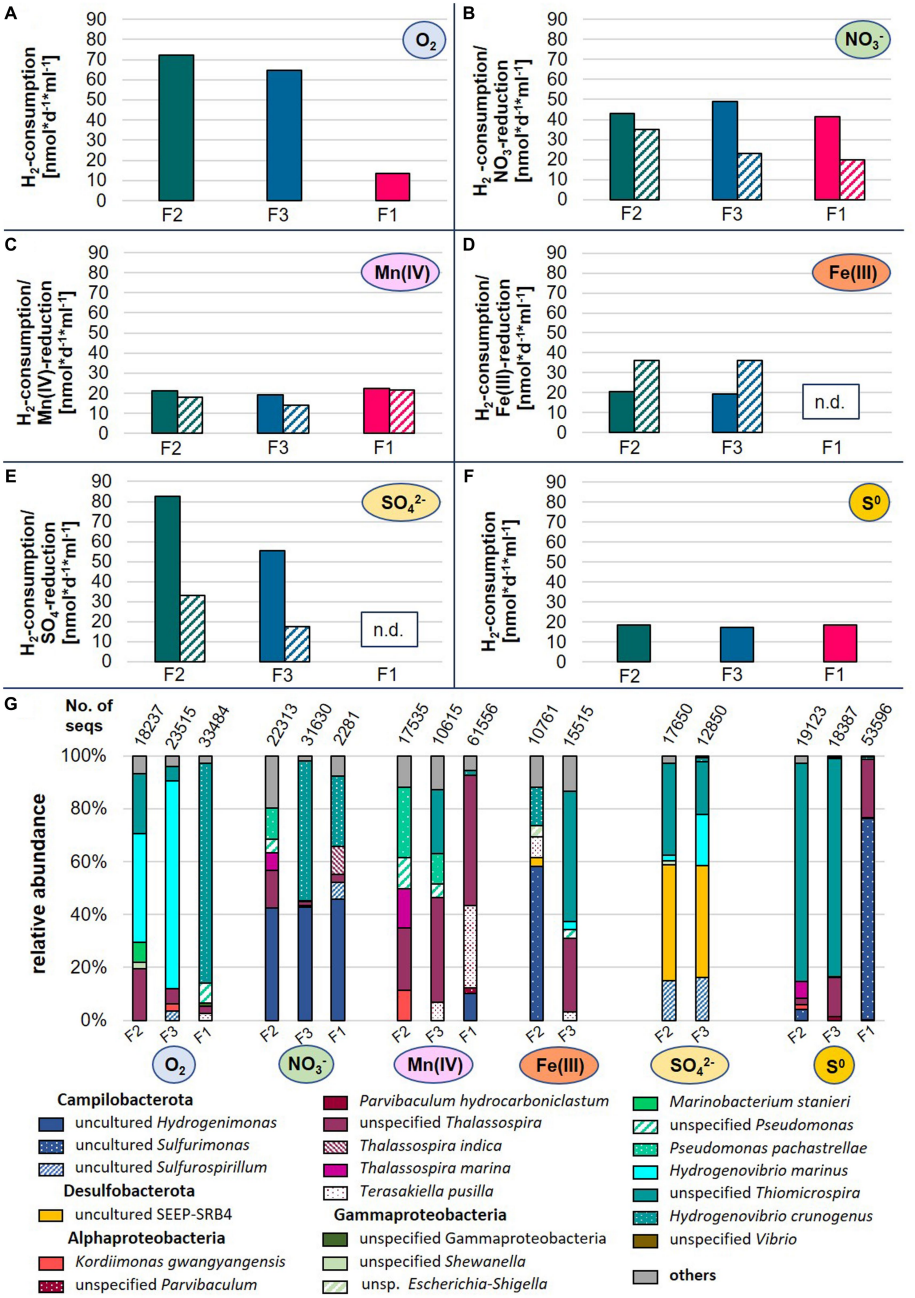
Hydrogen consumption rates **(A–F)** and microbial species abundances **(G)** of enrichment cultures inoculated with diffuse fluids. Hydrogen (filled bars) and electron-acceptor consumptions (dashed bars) are displayed as rates in nmol*d^−1^*ml^−1^ based on three technical replicates. For oxygen and elemental sulfur, no consumption rates are available. N.d., not detectable, No. of seqs denotes the number of merged sequences used for the calculation of relative abundances.

In hydrothermal vent environments reduced sulfur species (mainly hydrogen sulfide) and molecular hydrogen represent the most favorable electron donors for autotrophic growth of microorganisms (Adam and Perner, 2018). Given the high hydrogen concentrations measured in fluids from the Kairei vent field located on the CIR and previous isolations of hydrogen oxidizers from the CIR (Kumagai et al., 2008; Adam et al., 2021), we attempted to explore the hydrogen oxidation potential and electron-acceptor use of autotrophic microbial communities from three diffuse fluids (F2, F3, and F4) sampled within this study. After 1 year of consecutive transfers in organic-free artificial seawater medium with hydrogen as electron donor [H_2_/CO_2_ (80/20) atmosphere in the head space] and incubation at room-temperature, the five most abundant species of each culture constituted >80% of all species (including Archaea) observed in the respective communities (Figure 6G). In most cases, these species did not exhibit high abundances in the respective original fluids, likely resulting from the current inability to culture the microbial majority (Lloyd et al., 2018). Nevertheless, we were able to observe some sample-specific differences in consumption rates and compositions of the enrichments.

The highest hydrogen consumption rates of up to 83 nmol H_2_*d^−1^*ml^−1^ were observed for fluids F2 and F3 with oxygen or sulfate as electron donors (Figure 6). Still, these were in the lower range of rates previously measured in incubation experiments with hydrothermal fluids from the Mid-Atlantic Ridge (Perner et al., 2011). Overall, the enrichments of fluid F1 exhibited the lowest hydrogen consumption rates and with sulfate and Fe(III) as electron-acceptor, no growth and substrate consumption could be detected at all (Figure 6). This observation fits to the low abundance of typical chemoautotrophs in the bacterial communities of the original sample (*cf.* Supplementary Figure S1 and Table 6).

The use of oxygen as electron acceptor resulted in the dominance of *Hydrogenovibrio* and *Thiomicrospira* species in all enrichments (Figure 6G). In the past, *Thiomicrospira* species were mainly associated with sulfur oxidation. However, recent reclassifications placed several *Thiomicrospira* species into the *Hydrogenovibrio* genus and studies have shown the oxygen-coupled hydrogen oxidation ability of these organisms (Hansen and Perner, 2016; Gonnella et al., 2019). The nitrate-amended enrichments of F3 and F1 were also characterized by high proportions of *Hydrogenovibrio* members, accompanied by uncultured *Hydrogenimonas*. Members of this genus were also highly abundant in the corresponding F2 enrichment, and are known as nitrate-reducing hydrogen-oxidizers and have been isolated from vent environments (Takai et al., 2004). Surprisingly, the Mn(IV)- and Fe(III)-incubations did not result in the enrichment of typical dissimilatory metal-reducing hydrogen oxidizers (Figure 6G). Instead, we observed large proportion of heterotrophic Alphaproteobacteria (e.g., *Thalassospira* and *Parvibaculum*) (Rosario-Passapera et al., 2012; Liu et al., 2016) as well as *Hydrogenovibrio*, *Sulfuriomonas,* and *Pseudomonas* members in these enrichments. To our knowledge, none of the observed autotrophic hydrogen oxidizers (*Hydrogenovibrio* and *Sulfurimonas*) have so far been reported to use Mn(IV) or Fe(III) as electron acceptor. However, this may explain the comparably low hydrogen consumption rates of these incubations (Figure 6C,D). Despite the highest Fe-concentrations in the F1 fluid (Table 3), no Fe(III)-reducing culture could be enriched (Figure 6D). However, the comparably high manganese concentrations in F1 are reflected in the highest Mn(IV)-reduction rates among the enrichment cultures (*cf.* Table 3 and Figure 6C). Both sulfate-reducing incubations were dominated by the uncultured SEEP-SRB4 group. Although no cultured representative is available, these microbes are commonly associated with hydrocarbon-oxidation coupled to sulfate-reduction in hydrocarbon seeps and mud-volcanoes (Kleindienst et al., 2012). Thus, it is unlikely that they constitute the key hydrogen oxidizers in this incubation. Instead, we assume that this metabolism is carried out by members of the *Sulfurospirillum* genus, which constitute up to 16% and cultured representatives have been shown to couple hydrogen oxidation to sulfur reduction (Kodama et al., 2007). Still puzzling is the high abundance of *Hydrogenovibrio* members in these incubations (up to 42%), as they have not been reported to be involved in dissimilatory sulfate reduction. So far, only genes for the assimilatory reduction of sulfate have been detected in *Hydrogenovibrio* genomes (Jiang et al., 2017). One possible explanation for the presence of *Hydrogenovibrio* in these incubations is that the reduced sulfur compounds released by members of the highly abundant SEEP-SRB4 serve as an alternative energy source for *Hydrogenovibrio*. Elemental sulfur as electron acceptor led to the lowest hydrogen consumption rates of all incubations (Figure 6F). In the F2 and F3 incubations unspecified *Thiomicrospira* species dominated with proportions of 83% of all observed species (Figure 6G). Given the - for *Thiomicrospira* species - uncommonly low hydrogen consumption rates and their sulfur-oxidation ability, it could be assumed that these members used sulfur oxidation instead of hydrogen oxidation to support their growth. The same hypothesis could be postulated for the respective F1 incubation with regard to the dominance of the hydrogen-and sulfur-oxidizing *Sulfurimonas* genus. However, it was recently shown that several *Sulfurimonas* strains can couple the oxidation of hydrogen to the reduction of elemental sulfur (Wang et al., 2021).

### Correlations of microbial communities with environmental parameters and levels of hydrothermal activity

**Figure 7:**
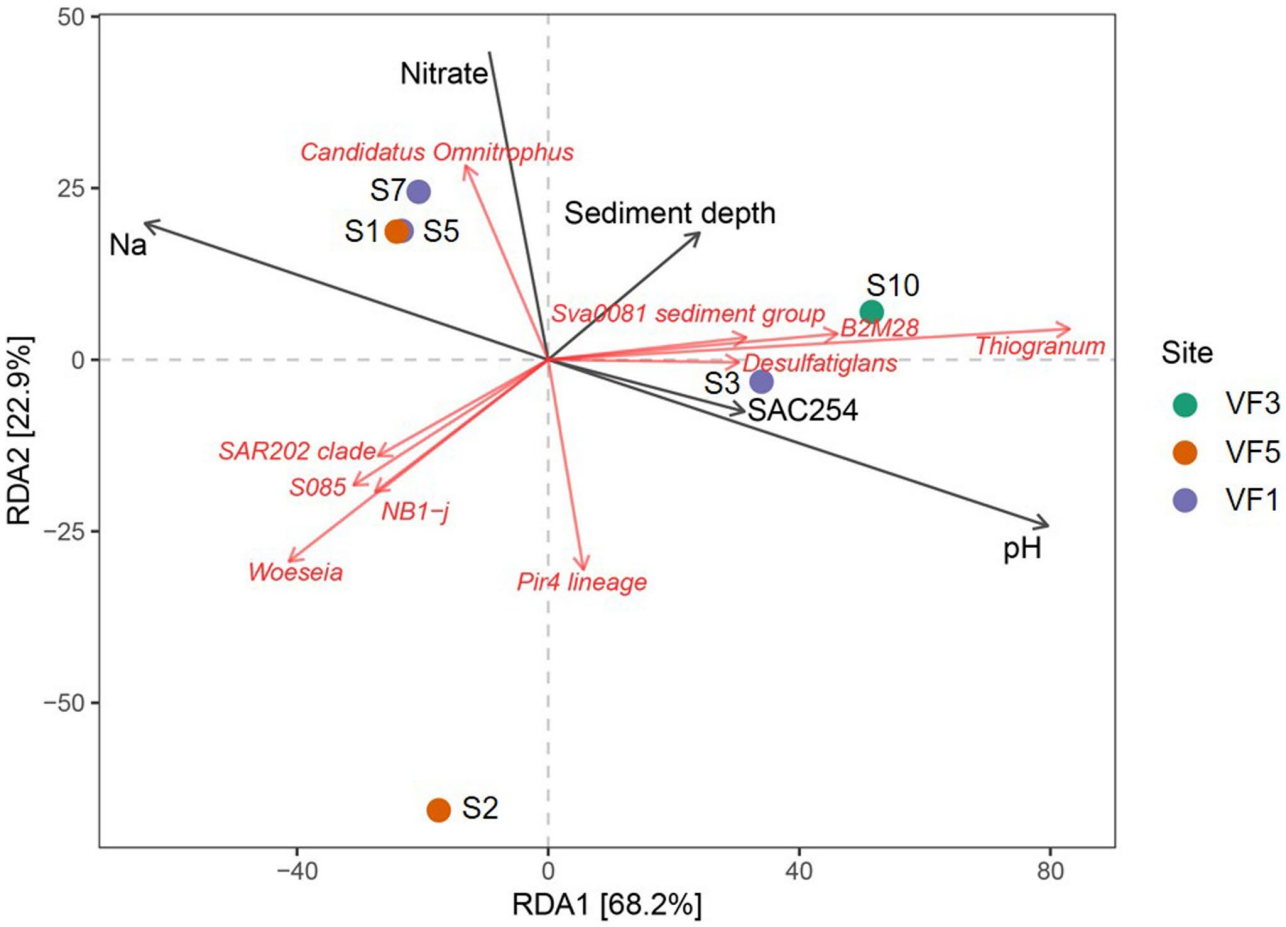
RDA plots showing correlations of environmental parameters with abundances of microbial taxa.

Due to the in most cases limited numbers of individual samples per sample type, statistically relevant correlations of microbial communities with environmental parameters could only be computed for the sediment samples of the INDEX2019 sampling campaign. For this purpose, we performed redundancy analyses of the bacterial taxonomies with the porewater data. In the corresponding ordination plot (Figure 7) 68% (RDA1) and 23% (RDA2) of the variation was explained by the first two axes. Strong negative correlations were observed between the sediment depth and the abundance of Bacteria belonging to the SAR202 clade, S085 and NB1-j groups, *Woeseia* genus and the Pir4-lineage (Figure 7). Except for the Pir4-lineage, these groups and genera also showed a weak correlation with sodium. Nitrate concentrations correlated positively with *Candidatus* Omnitrophus, while a negative correlation was observed with the Pir4-lineage. For *Candidatus* Omnitrophus various metabolic traits have been inferred from metagenomic analyses, including heterotrophy, autotrophy, methane oxidation, and also nitrate reduction (Rinke et al., 2013; Momper et al., 2017). Given the correlations and relative bacterial abundances of up to 5% for *Candidatus* Omnitrophus, nitrate reduction may play a significant role in the VF 5 surface sediments (S1 and S2) and the 4–8 cm horizons of VF 1 sediments (S5 and S6). Furthermore, members of the metabolically diverse Sva0081 sediment group, B2M28 group and *Thiogranum* genera showed a rather weak positive correlation with sediment depth. Strong positive correlations with pH and SAC254 were observed for the *Desulfatiglans* genus (Figure 7). SAC254 displays a parameter indicating the presence and concentration of certain organic substances (above all aromatic compounds and humic acids). The genetic potential for the utilization of aromatic compounds found in some *Desulfatiglans* strains might explain this correlation (Jochum et al., 2018). In contrast to our observations from the INDEX2016 sampling campaign of Kairei sediments (Adam et al., 2019), in our current study no correlations between metal concentrations and bacterial communities or individual taxa was observed.

When considering whole bacterial communities of the sediment cores, correlations with sodium and nitrate were visible for two sediment horizons of VF 1 as well as the bulk sediment sample from the VF 5 vent site. Interestingly, the surface sediment horizon from VF 1 showed correlations with SAC254 and pH, rather than sodium and nitrate, matching the outlier position of this sediment horizon in the PCoA-plots (*cf.* Figures 4A,B,7). The 10–12 cm sediment horizon from VF 3 harbored the only bacterial community correlating with sediment depth, shaped by the above-mentioned correlating lineages. The greatest distance to other communities and environmental factors in the RDA-plots was observed for the surface sediment S2 from the VF 5 vent site (Figure 7). Against our expectations, no correlations with the distance to the next active venting site were observed, indicating that the geochemical parameters shaping the sediment communities are not directly linked to the current hydrothermal activities. This hypothesis is also supported by the lack of typically vent-associated chemoautotrophs in the studied sediment communities, even though the respective geochemical analyses identified hydrothermal sediment input at least in the surface horizons of the sampled sediments.

The comparably low bacterial diversity and predominant absence of chemosynthetic Bacteria, especially from the *Campylobacterales* order, in the diffuse fluid from VF 4 (F1) cannot be fully explained by the respective fluid geochemistry. However, the absence of sulfide species (below detection limit, Table 3) indicates that an important electron-donor for microbial vent communities (Perner et al., 2007; Amend et al., 2011) is missing in this habitat. The comparably low hydrogen oxidation potential in the corresponding enrichment cultures and absence of typical hydrogen oxidizers in the original communities further suggest that the hydrogen concentrations in fluid sample F1 are not sufficient to support a rich chemosynthetic community. Apart from this, the mixing with ambient seawater, characteristic for diffuse fluids, may also entrain seawater communities to the diffuse fluids (e.g., Anderson et al., 2013). The exceptionally high abundance of the *Alcanivorax* genus in this sample may indicate the presence of volcanic activity related hydrocarbon leakage in the vicinity of the fluid emission as recently hypothesized for *Alconivorax*-dominated hydrothermal plumes in the South-Pacific Ocean (Dede et al., 2023). Further indication for such a leakage may arise from the high abundance of methylotrophic taxa in the microbial mat sampled at VF 4. Although we unfortunately have no data on hydrocarbon concentrations in the sampled fluids, it can be assumed that fluid F1 contained higher fractions of H_2_ and methane (CH_4_). As vent field VF 4 is placed in an ultramafic rock-hosted setting, the associated serpentinization most likely led to high H_2_ concentrations. The excess of H_2_ can in turn produce CH_4_ and other hydrocarbons (e.g., by Fischer-Tropsch reactions). Similar processes have been observed before in the Rainbow vent field at the MAR (Charlou et al., 2002). In the vicinity of the sampling site of diffuse fluid F1, high-temperature fluid emissions were also observed. Given the – compared to seawater – high heavy metal concentrations in this fluid (Table 3), elevated concentrations of lead, cadmium, zinc, manganese and iron can also be expected to prevail in the resulting plume and surroundings. This might further contribute to the high abundances of the *Alcanivorax* genus, which previously demonstrated the ability for the detoxification of heavy metals in polluted sediments (Zhong et al., 2020).

Corresponding to the predominantly low Fe-contents in the sampled fluids, no Fe-oxidizing Bacteria and Archaea were detected in the respective communities. The occurrence of common Fe-oxidizing microbes (i.e., *Mariprofundus* species) was limited to the microbial mat samples M1-M3 (Edmond vent field, VF3 and VF4) as well as the clogged sulfide chimney R2 (VF1), indicative of previous Fe-rich venting at the respective sites. Since its first description in 2007, the *Mariprofundus* genus has frequently been identified in iron-rich hydrothermal vent environments and microbial mats, also from the SWIR, and are hypothesized to play an important role in the formation of Fe-oxyhydroxides in vent environments (Emerson et al., 2007; Singer et al., 2011; Li et al., 2013; Zhong et al., 2022). However, we did not observe higher abundances (i.e., >5%) of *Mariprofundales* in the microbial mat and sulfide samples of the Kairei and Pelagia vent fields in our previous study (Han et al., 2018). Unfortunately, we do not have any mineralogical or geochemical data on the host rocks of our microbial mat samples, but visual inspections of the sampling sites (Figure 2) already suggested the presence of Fe-oxides. Hence, Fe-oxidation appears to play a major role for energy conservation in the respective microbial mats. The mineralogical profile of the Fe-encrusted clogged sulfide chimney R2 was characterized by the highest concentrations of chalcopyrite across all samples of our 2019 sampling campaign, which may serve as a rich, biologically available Fe-, H_2_S, and S-source and results in the high abundances of *Mariprofundales* and sulfur-oxidizing Gammaproteobacteria as described above.

The distinct geochemical characteristics of VF 2, i.e., the presence of silica mounds and granules with minor proportions of pyrite as well as the occurrence of Fe-depleted fluids coincides with exceptionally high abundances of uncultured genera, which in part were only found in the microbial community of the sampled precipitates. These included bacterial groups associated with nitrogen and carbon cycles, such as uncultured, putatively nitrifying *Nitrospina* and *Nitrosomonas* species (Koops and Pommerening-Röser, 2001; Lücker et al., 2013), uncultured chemoheterotrophic SAR202 Chloroflexi (Mehrshad et al., 2018) as well as putatively denitrifying, uncultured members of the *Kiloniellales* (Wiese et al., 2020). Some of these “VF 2-exclusive” were also identified in sediments from the Kairei vent field and already showed significant correlations with the silicate phases of the respective sediments (Adam et al., 2019), which gives further indications for the ecological importance in the silica-rich VF 2.

### Putative ecosystem functions and impacts of mining scenarios

In addition to common heterotrophs, autotrophic microbial taxa and groups were observed in all sample and habitat types (*cf.* Supplementary Figure S1 and Tables 4–6). Given the relative abundances of these bacterial and archaeal autotrophs in the specific habitats (Supplementary Figure S1), the expected primary biomass production is generally higher in the actively venting environments compared to those currently missing hydrothermal activity and especially the sampled sediments. This microbial primary production serves as the basis for the rich fauna at active venting sites and thus contributed significantly to the demands for the protection of active hydrothermal vents (e.g., van der Most et al., 2023). However, the microbial mats, sampled from non-venting sites of different vent fields, with their noticeable high numbers of autotrophic *Mariprofundales* (Table 4; Singer et al., 2011) suggest that the microbial potential for CO_2_-fixation and primary biomass production in non-venting habitats should not be neglected when assessing the possible ecological impacts of SMS mining. Given the fact that all our samples were taken from vent fields that still harbor at least a few actively venting sites, the microbial community compositions and primary production potential of fully extinct deep-sea hydrothermal vent sites may differ considerably. The microbial communities of the sampled massive sulfides, rocks and microbial mats also appear to be involved in various biogeochemical cycles, especially the nitrogen and sulfur cycles as discussed above. As we observed before in the 2016 sampling campaign, the high numbers of uncultured or unspecified microbes make it difficult to assess the impacts of the removal as well as the resilience of the respective communities and/or habitats (Han et al., 2018).

Apart from the putative removal of ecologically relevant microbial communities, deep-sea mining may also impact the environments by sediment/waste plumes generated in the mining process and expected to be emitted close to the seabed (Amon et al., 2022). Depending on the plume composition and deposition site of the sedimentary freight, different effects can be expected: if hydrothermal input accumulated on the sediments sampled during our 2019 campaign, a community shift with the enrichment of typically vent-associated microbes may occur. How persistent such a shift would be is questionable, since a constant hydrothermal fluid input is missing. In case of a high (heavy) metal load, toxic effects may also alter the benthic microbial populations and their respective biogeochemical cycling capacities, favoring the enrichment of putatively detoxifying (metal-reducing or-precipitating) species such as *Alcanivorax* (Zhong et al., 2020), which was highly abundant in one of our fluid samples (Table 6). However, an overall loss of microbial biodiversity on the seafloor habitats sampled within this study cannot be excluded as a possible effect of such a mining plume (*cf.* Jones et al., 2018; Amon et al., 2022).

## Conclusion

As the interest in the mining of SMS deposits and the development of respective mining technologies are continuously increasing, the environmental impact assessment of mining activities is of fundamental relevance to develop mitigation strategies and foster a resilient ocean. With this study we expand the baseline knowledge about microbial communities as an integral part of ecosystems from Indian Ridge venting environments, including exceptional hydrothermal vent systems like VF 4. Given the overall high numbers of uncultured and unspecified microbes and the lack of typical community distribution patterns (e.g., zonation along distances to the active vents) in these venting environments, forecasting of mining impacts and the resilience of microbial communities remains difficult. Thus, continuous efforts in the study of microbial ecosystem functions in hydrothermal vent environments and SMS deposits are of eminent importance and must precede any disturbances of these ecosystems.

## Data availability statement

The datasets presented in this study can be found in online repositories. The names of the repository/repositories and accession number(s) can be found at: https://www.ncbi.nlm.nih.gov/bioproject; PRJNA951358.

## Author contributions

MP and AS planned the study. MP and NA-B assessed the compiled data and prepared the manuscript outline. NA-B wrote the manuscript with contributions and approval of all authors. DG-S performed fluid sampling and geochemical analyses of hydrothermal fluids. SF was responsible for mineralogy and geochemistry. SP contributed to the evaluation of geological data. KL-M collected samples for microbiological analyses, set up microbial enrichment cultures, measured consumption rates, extracted DNA and prepared 16S amplicons. DI performed the Illumina Sequencing. NA-B and KL-M maintained the enrichment cultures, performed bioinformatic and statistical analyses and evaluated the data. All authors contributed to the article and approved the submitted version.
