# Supplementary material for: Microbial ecosystem assessment and hydrogen oxidation potential of newly discovered vent systems from the Central and South-East Indian Ridge
Source: Front Microbiol. 2023 Oct 11;14:1173613. doi: 10.3389/fmicb.2023.1173613 (PMC10598711; doi:10.3389/fmicb.2023.1173613)

**Supplementary information for:**

**Microbial ecosystem assessment and hydrogen oxidation potential of newly discovered vent systems from the Central and South-East Indian Ridge**

Nicole Adam-Beyer^1^*, Katja Laufer-Meiser^1^, Sebastian Fuchs^2^, Axel Schippers^2^, Daniela Indenbirken^3^, Dieter Garbe-Schönberg^4^, Sven Petersen^5^, and Mirjam Perner^1^*

^1^ Marine Geosystems, GEOMAR Helmholtz Centre for Ocean Research Kiel, Wischhofstr. 1-3, 24148 Kiel, Germany

^2^ Federal Institute for Geosciences and Natural Resources (BGR), Stilleweg 2, 30655 Hannover, Germany

^3^ Leibniz Institute for Virology, Martinistraße 52, 20251 Hamburg, Germany

^4^ Institute of Geosciences, Christian-Albrechts-Universität zu Kiel, Ludewig-Meyn-Straße 10, 24118 Kiel, Germnay

^5^ GEOMAR Helmholtz Centre for Ocean Research Kiel, Wischhofstr. 1-3, 24148 Kiel, Germany

* Corresponding authors: Mirjam Perner, E-mail mperner@geomar.de and Nicole Adam-Beyer, E-mail nadam@geomar.de

Key words: 16S rRNA, geochemistry, hydrothermal vents, sediments, Indian Ridge

This file contains Supplementary Figure 1

Not included in this document: Supplementary Tables 1 and 2

**Supplementary Figure 1:** Taxonomy plots of Bacteria and Archaea. Proportions of 16S rRNA gene tags related to major bacterial (A) and archaeal (B) orders for the analyzed samples of the INDEX2019 sampling campaign. Only bacterial groups with at least 5% abundance in one of the samples are shown. Low abundant orders of the major phyla are summarized as “other” (e.g. other Bacteroidota), while “low abundance” refers to low abundant orders of phyla not represented in the plot. For the archaeal communities no cut-off was used. No. of seqs denotes the number of merged sequences used for the calculation of relative abundances.


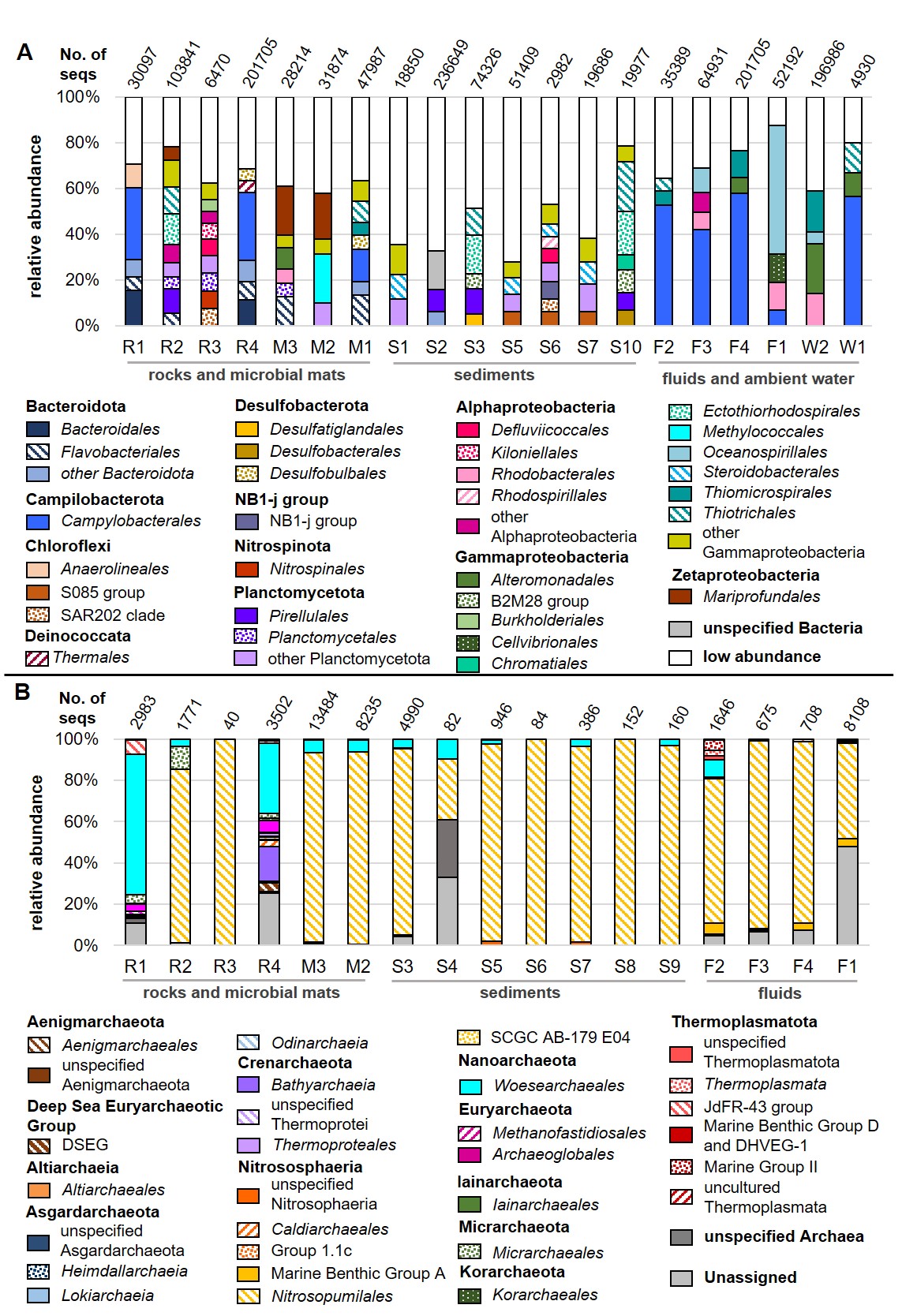

Supplement: Supplementary file 1 [file Data_Sheet_1.DOC]
